# Induction of tier-2 neutralizing antibodies in mice with a DNA-encoded HIV envelope native like trimer

**DOI:** 10.1038/s41467-022-28363-z

**Published:** 2022-02-04

**Authors:** Ziyang Xu, Susanne Walker, Megan C. Wise, Neethu Chokkalingam, Mansi Purwar, Alan Moore, Edgar Tello-Ruiz, Yuanhan Wu, Sonali Majumdar, Kylie M. Konrath, Abhijeet Kulkarni, Nicholas J. Tursi, Faraz I. Zaidi, Emma L. Reuschel, Ishaan Patel, April Obeirne, Jianqiu Du, Katherine Schultheis, Lauren Gites, Trevor Smith, Janess Mendoza, Kate E. Broderick, Laurent Humeau, Jesper Pallesen, David B. Weiner, Daniel W. Kulp

**Affiliations:** 1 Vaccine and Immunotherapy Center, The Wistar Institute, Philadelphia, PA 19104 USA; 2 Perelman School of Medicine, University of Pennsylvania, Philadelphia, PA 19104 USA; 3 Inovio Pharmaceuticals, Plymouth Meeting, PA 19462 USA; 4 Molecular and Cellular Biochemistry, Indiana University, Bloomington, IN 47405 USA

**Keywords:** DNA vaccines, Cryoelectron microscopy, HIV infections

## Abstract

HIV Envelope (Env) is the main vaccine target for induction of neutralizing antibodies. Stabilizing Env into native-like trimer (NLT) conformations is required for recombinant protein immunogens to induce autologous neutralizing antibodies(nAbs) against difficult to neutralize HIV strains (tier-2) in rabbits and non-human primates. Immunizations of mice with NLTs have generally failed to induce tier-2 nAbs. Here, we show that DNA-encoded NLTs fold properly in vivo and induce autologous tier-2 nAbs in mice. DNA-encoded NLTs also uniquely induce both CD4 + and CD8 + T-cell responses as compared to corresponding protein immunizations. Murine neutralizing antibodies are identified with an advanced sequencing technology. The structure of an Env-Ab (C05) complex, as determined by cryo-EM, identifies a previously undescribed neutralizing Env C3/V5 epitope. Beyond potential functional immunity gains, DNA vaccines permit in vivo folding of structured antigens and provide significant cost and speed advantages for enabling rapid evaluation of new HIV vaccines.

## Abstract (web summary)

HIV envelope is a target for vaccine development, but induction of broadly neutralizing antibodies has been difficult. Here, the authors show that electroporation with a synthetic DNA vaccine construct allows in vivo production of HIV envelope native like trimers to induce autologous tier-2 neutralizing antibodies targeting a C3/V5 epitope in mice.

## Introduction

The sequence diversity and unique glycosylation profiles of the HIV-1 surface protein, Envelope (Env), hamper the development of an efficacious prophylactic HIV-1 vaccine that induces broadly neutralizing antibody (bNAb) responses^1,2^. The early generations of HIV-1 vaccines included the monomeric gp120 domain or non-native gp140/160 forms of Env. Though these vaccines induced binding antibodies which recognized Env, the responses were not able to neutralize circulating HIV-1 viruses^3,4^. Over the years, various modifications to the Env protein were explored to create trimeric proteins. Early iterations included the use of foldon or isoleucine zipper domains to form non-native trimer structures^5,6^. In recent years, several modifications have been found to allow the folding and production of trimer proteins that resemble the native viral Env conformations, or native-like trimers (NLTs)^7–11^. Biophysical techniques such as cryo-electron microscopy (cryo-EM) and X-ray crystallography of various identified broadly neutralizing antibodies (bNAbs) bound to NLTs confirmed that these NLTs can present important conformational neutralizing epitopes in a solvent-accessible fashion^8,12^. Importantly, when rabbits and non-human primates (NHPs) were immunized with adjuvant co-formulated protein NLTs, autologous tier-2 neutralizing antibodies were induced and the responses from a select group of vaccinated animals were able to protect some NHPs from an autologous tier-2 SHIV challenge^13–15^.

However, experiments using rabbits and macaques are costly and lack throughput. For many infectious diseases, mice have been identified as an ideal small laboratory animal for preliminary screening and down-selection of designed immunogens, prior to evaluations of the promising clinical candidates in larger animals^16^. In the case of HIV-1, however, it has been demonstrated that induction of autologous Tier-2 neutralizing antibody responses with protein NLTs in mice were extremely difficult, regardless of the choice of mouse strains, adjuvants, or routes of vaccination (subcutaneous depot or slow-release mechanism using an osmotic pump)^17,18^. Several hypotheses were proposed for these findings, namely the immune-dominance of epitopes at the base of soluble NLTs which are not normally exposed in native virions, as well as deficiencies in mouse antibody repertoire to recognize glycan-containing epitopes^8,17^. Many subsequent studies with protein NLTs were, therefore, not performed in mice and advanced directly for evaluations in rabbits and macaques^19–21^, even though it has been shown in a more recent study that scaffolding of NLTs in a multi-valent fashion on nanoparticle scaffolds may facilitate the induction of autologous Tier-2 antibody responses in mice^22^.

DNA vaccines are an approach where engineered nucleic acids are administered to a host for in vivo production of an antigen to elicit immune responses^23^. While early studies demonstrated the immunogenicity of DNA vaccines in larger animals and humans was sub-optimal, advances in antigen design, nucleic acid formulations, genetic adjuvants, and adaptive electroporation (EP) technologies have now enabled DNA vaccines to induce more potent and consistent responses in several clinical studies, including Zika, MERS and Ebola^24–26^. In the HIV space, a DNA vaccine encoding two consensus Envs, Gag, and Pol antigens adjuvanted with plasmid IL-12 delivered by intradermal electroporation induced 98% seroconversion, tier 1 neutralization, and strong cellular responses in the HVTN098 study^27^. Importantly, we have also demonstrated that DNA/EP can be used for direct in vivo delivery of highly-folded monoclonal antibodies and biologics^28–30^, and to launch de novo assembly of more complex nanoparticle vaccines in the hosts to induce immune responses phenotypically different from protein nanoparticle vaccines^31^.

In this work, we attempted to determine if DNA/EP can be used for in vivo expression of a conformationally complex NLT and, if so, characterize immune responses induced by DNA versus protein NLT vaccinations using previously well-characterized stabilized NLT BG505.MD39 (Stabilized HIV Env Gp140 ectodomain) as a model antigen^32^. We observed DNA/EP resulted in direct in vivo production and proper assembly of NLTs using blue NATIVE PAGE analysis as well as staining of tissues with bNAbs and non-nAbs. Comparison of protein versus DNA MD39 vaccinations in mice demonstrated induction of trimer-binding antibodies by both routes. However, CD4 + and CD8 + T-cell responses, as well as autologous Tier-2 BG505.T332N neutralization was observed in mice immunized with DNA but not protein MD39. T-cell and neutralizing antibody (nAb) responses induced by DNA NLT were found to be further improved by increasing the interval between vaccinations. We further characterized the nAbs by engineering pseudovirus variants with modified glycosylation sites and single-cell RNA sequencing on sorted antigen-specific B-cells. Five unique murine neutralizing antibody clones were isolated, and one was characterized with cryo-EM to atomic resolution and found to be specific for a neutralizing epitope on the V5 loop of the Env trimer. This study demonstrated that DNA/EP can be used for direct in vivo expression of conformationally complex antigens such as NLT and can elicit phenotypically different immune responses, particularly nAb responses, consistently in animals. Further studies to compare these two routes of immunizations and to explore the unique properties of DNA vaccines such as inducing T-cell and nAb responses are likely important for HIV-1 and other infectious diseases.

## Results

### Synthetic DNA (synDNA) encoded native-like trimers properly fold, express, and maintain an antigenic profile in vitro and in vivo

**Fig. 1:**
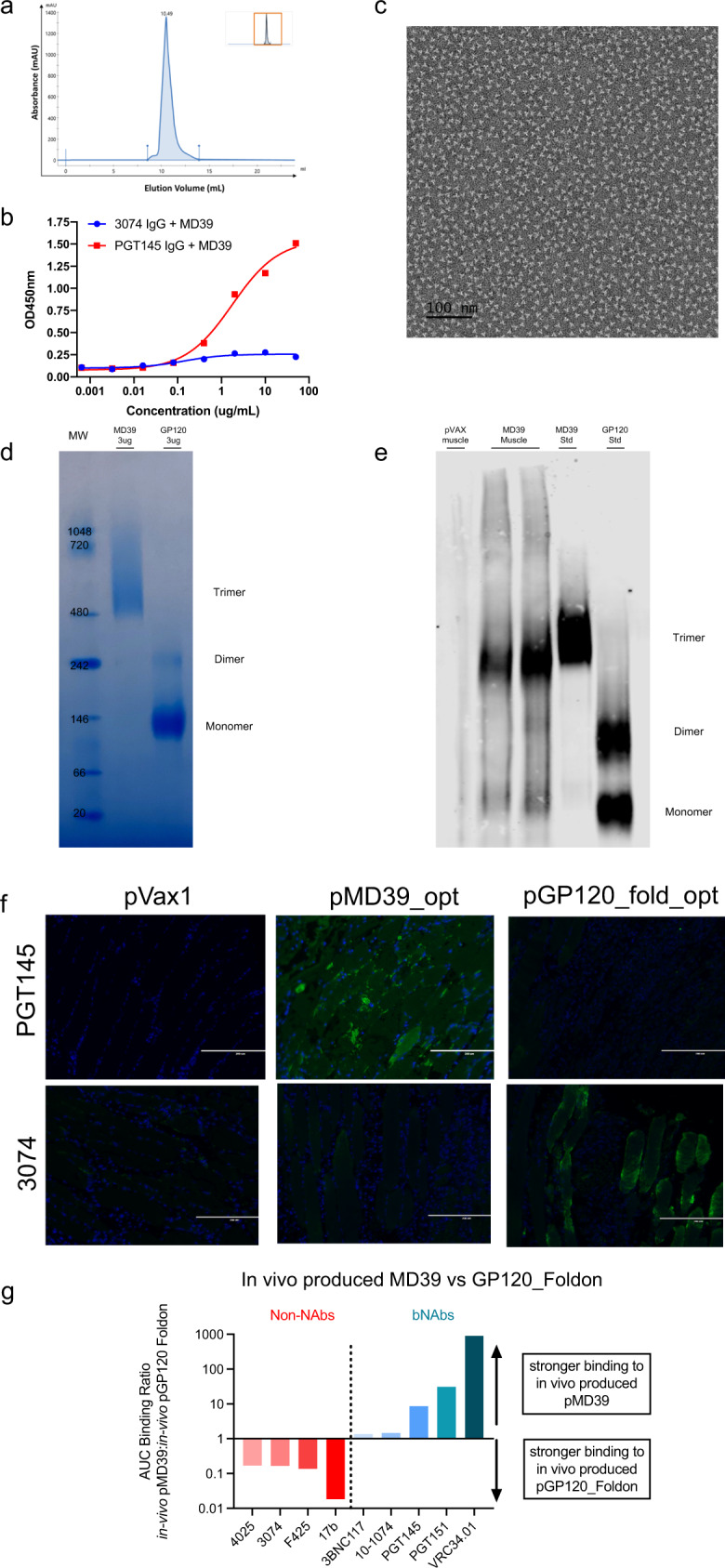
DNA/EP delivery of BG505.MD39 can enable in vivo expression of conformationally complex NLT. **a** SEC trace of lectin-column purified BG505.MD39 that has been sequence optimized for in vivo expression. **b** Antigenic profile of sequence optimized BG505.MD39 in terms of ELISA binding to V2-apex directed bNAb PGT145 and v3-directed non-nAb 3074. **c** nsEM image of SEC purified sequence optimized BG505.MD39. **d** Coomassie staining for the migrations of the purified in vitro expressed BG505.MD39 and BG505 gp120 protein standards on 3–12% Bis-Tris NATIVE PAGE gels. **e** 2G12-based western analysis to compare the migrations of the in vivo produced BG505.MD39 in muscle homogenates from mice treated with DNA-encoded BG505.MD39 (harvested 4 d.p.i) to those of in vitro produced BG505.MD39 and BG505 gp120 on a 3–12% Bis-Tris NATIVE PAGE gel. **f** Immunofluorescence analyses of muscle sections from mice treated intramuscularly with DNA-encoded BG505.MD39 or BG505.gp120.foldon (harvested 7 d.p.i) in terms of binding to bNAb PGT145 or non-nAb 3074. **g** Normalized area under the curve (ratio of in vivo produced pMD39 binding versus pGP120-foldon binding) for binding to HIV bNAbs and non-NAbs. For **c**, **d**, **e**, and **f**, the experiments were repeated twice.

We optimized in vivo expression of BG505.MD39 by performing codon and RNA optimization of the antigen nucleotide sequence and including an N-terminal IgE leader sequence which facilitated both antigen trafficking to the secretory pathway and ribosomal loading^28,33^. To ensure intact biophysical and antigenic profiles for the optimized BG505.MD39, we expressed the construct in vitro using Expi293F cells. Size Exclusion Chromatography demonstrated homogeneous assembly of lectin purified BG505.MD39 which eluted as a single fraction that corresponds to a trimer (Fig. 1a). Using binding ELISA, expressed BG505.MD39 was shown to bind to bNAb PGT145, which preferentially recognizes the trimeric conformational epitope on Env V2-apex^34^, but not non-NAb 3074, which is specific for the Env V3 loop which is an epitope exposed on monomeric gp140 but hidden in a NLT^35–37^ (Fig. 1b). A full antigenic profile of the optimized BG505.MD39 is consistent with previous studies (Supplementary Fig. 1A–C)^8,32^. Negative-stain Electron Microscopy (nsEM) demonstrated intact trimeric propeller-shaped conformation of BG505.MD39^15^ (Fig. 1c).

We characterized the biophysical and antigenic profiles of DNA-delivered in vivo produced NLTs. To our knowledge, this type of in vivo profiling of NLTs has not been previously studied. Muscle homogenates of BALB/c mice intramuscularly inoculated with plasmid BG505.MD39 (pMD39_opt) were obtained 4 days post-injection (d.p.i). We first visualized how purified BG505.MD39 and recombinant BG505.gp120 proteins would migrate on a blue NATIVE PAGE gel using Coomassie staining (Fig. 1d). While analysis of glycoproteins with PAGE is challenging, we observed BG505.MD39 standard migrated as a trimer at a molecular weight (MW) of ~480 kDa. In comparison, BG505.gp120, as expected, migrated mainly as a monomer, and a small fraction as a dimer, on the NATIVE PAGE gel at MWs of ~120 and ~240 kDa, respectively. As muscle homogenates contain proteins other than BG505.MD39, we used 2G12-based Western analysis to more specifically detect bands that correspond to HIV Env proteins. We observed that BG505.MD39 in muscle homogenates migrated mainly as trimers in blue NATIVE PAGE gel in comparison to the protein BG505.MD39 standard, with small fractions existing as monomers or dimers as is consistent with domain-wise global folding pathways of newly synthesized polypeptides^38^ (Fig. 1e). Differences in vivo produced versus recombinantly produced BG505.MD39 may reflect differences in glycosylation profiles between Exp293F cell line versus murine muscle cell-produced glycoproteins. To further assess the antigenic profile including glycan-sensitive antibodies of in vivo produced versus recombinant NLTs, we have developed and performed Antigen Conformation Tracing In Vivo by ELISA (ACTIVE) to compare in vivo produced versus recombinant MD39 or gp120-foldon in binding to HIV antibodies. We observed that in vivo produced pMD39, as compared to pGP120-foldon, was capable of binding more strongly to V3-glycan specific antibody 10–1074^39^, trimer apex glycan specific antibody PGT145^40^, and gp41 glycan specific antibody PGT151^41^ (Supplementary Fig. 1D).

A critical characteristic of a vaccine immunogen is the retention of proper conformation in vivo. We developed an experiment to assess in vivo antigenic profiling by immune-fluorescence (IVAP). Briefly, BALB/c mice are injected with DNA plasmids, and muscles are harvested seven d.p.i and stained with bnAbs or non-nAbs, followed by fluorophore-conjugated anti-human secondary antibody. We conducted an IVAP experiment with an empty backbone (pVAX1), pMD39_opt, or pGP120_foldon (a non-native-like trimer of gp120s) (Fig. 1f). Muscle injected with pMD39_opt had strong staining for trimer-specific bnAbs (PGT145) but not a V3-directed non-nAbs (3074). In contrast, muscles injected with pGP120_foldon had strong staining with 3074 and not PGT145. The IVAP analysis demonstrated DNA-delivered NLTs express well-folded trimeric immunogens, supporting the use of DNA/EP for delivery of conformationally complex HIV immunogens. To further validate the antigenic profile of in vivo produced materials, we analyzed in vivo produced NLT versus gp120-foldon in binding to more trimer-specific antibodies including bNAbs (PGT145, PGT151, VRC34.01) and monomer-specific non-NAbs (3074, F425,4025, 17b) with our ACTIVE protocol (Fig. 1g). We observed overall binding profiles of in vivo produced MD39 to the panel of bNAbs and non-nAbs behaved very similarly compared to recombinant MD39 (Supplementary Fig. 1F), with in vivo produced pMD39 preferentially binds to trimer-specific antibodies, whereas in vivo produced pGP120-foldon bound to monomer-specific antibodies. In addition, we can deduce that in vivo produced pMD39 is properly cleaved, as it is capable of binding to cleavage-dependent trimer-specific antibody VRC34.01^42^.

### DNA-encoded NLT immunization induced stronger T-cell and nAb responses as compared to corresponding protein immunization in mice

**Fig. 2:**
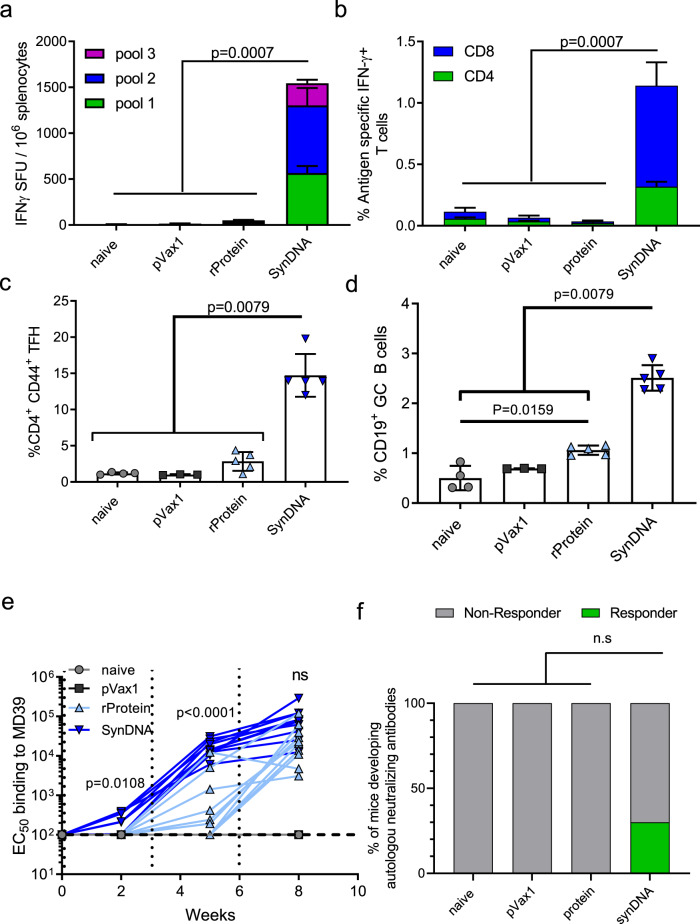
DNA immunization of BG505.MD39 induced stronger T-cell responses and NAb responses than protein immunization in BALB/c mice. All mice in this panel were immunized at Wks 0, 3, 6 with 25 ug DNA or 25 ug RIBI-co-formulated protein and euthanized 2 weeks post the final vaccination for cellular analyses. **a**, **b** Comparison of BG505 Env-specific cellular responses in naïve mice, or mice immunized with pVAX plasmid backbone, RIBI-co-formulated protein BG505.MD39, or DNA-encoded BG505.MD39 by IFNγ ELIspot assay (**a**) or intracellular cytokine staining (**b**). **c** Frequencies of CXCR5 + PD1 + Tfh cells amongst CD4 + CD44 + cells in the draining lymph nodes in naïve mice or 10 days post pVAX1, protein MD39 or DNA BG505.MD39 immunization. **d** Frequencies of GL7 + GC B cells amongst CD19 + B cells in the draining lymph nodes in naïve mice or 10 days post pVAX1, protein BG505.MD39 or DNA BG505.MD39 immunization. **e** Trimer-specific binding antibody responses induced in mice vaccinated with protein or DNA-encoded BG505.MD39 post the first, second and third immunization as determined by ELISA. **f** Frequencies of mice that developed autologous BG505.T332N neutralizing antibodies (ID_50_ titers greater than 1:45) in naïve mice or mice immunized with pVAX1, protein BG505.MD39 or DNA-encoded BG505.MD39 two weeks post the final vaccination. The dashed vertical line in **e** refers to the immunization timepoints. Two independent experiments were performed for each panel in the figure. *N* = 10 mice/group for protein or DNA-encoded BG505.MD39, *N* = 5 mice/group for naïve mice or pVAX1 treated mice (**a**, **b**, **e**, **f**); *N* = 5 mice/group (**c**, **d**); each dot or line represents an animal. Error bar represents standard deviation. Center of the error bar represents the mean. Two-tailed Mann–Whitney Rank test used to compare groups; *p*-values were adjusted for multiple comparison for panels (**a**–**d**). Two-tailed Mann–Whitney Rank tests were used to compute the *p*-values comparing EC50 titers between protein and DNA groups for each specific timepoint in **e**. Two-sided Fisher Exact Tests were used to compare proportion of responders between SynDNA group and naïve/ pVAX1/ protein groups in **f**. ns not significant. Source data are provided as a Source Data file.

We next compared immune responses induced by protein and DNA-encoded BG505.MD39 in mice.BALB/c mice were immunized with the same dose (25ug) of either DNA-encoded or RIBI formulated protein BG505.MD39 delivered at weeks 0, 3, and 6. Two weeks after the final immunization, mice were sacrificed, and cellular responses were determined using 15mer overlapping peptides that span the wildtype (WT) BG505 Envelope. As measured by the IFNγ ELISpot assay, mice vaccinated with pMD39_opt developed significantly stronger Env-specific T-cell responses than mice immunized with protein BG505.MD39 (Fig. 2a). These antigen-specific cellular responses generated recall from peptides spanning multiple regions of Env. Importantly, both CD4^+^ and CD8^+^ T-cell responses were induced by DNA vaccination (Fig. 2b), and antigen-specific CD4^+^ and CD8^+^ T cells exhibited significant poly-functionality in terms of simultaneous expression of IFN-γ, TNFα, and IL-2 (Supplementary Figs. 2, 3A-B). Activation of both T follicular helper (Tfh) cells and germinal center (GC) B cells has been demonstrated to be instrumental to the induction of humoral responses^43,44^. Here, we characterized the kinetics of Tfh and GC B-cell responses in muscle draining lymph nodes and spleen following EP-mediated pMD39_opt delivery. It was observed that the frequency of CD4^+^CD44^+^PD1^+^CXCR5^+^Tfh cells peaked around 7 d.p.i whereas frequency of CD19^+^GL7^+^ GC B cells increased past 10 d.p.i in the draining lymph nodes^45^ (Supplementary Fig. 4A, B). However, we did not observe increased frequency in either population in the spleen. Next, we compared Tfh and GC B-cell responses in mice immunized with either DNA-encoded or RIBI-co-formulated protein BG505.MD39 in the draining lymph nodes 10 d.p.i, and observed increased frequencies in Tfh and GC B cells in mice immunized with DNA versus those immunized with protein (Fig. 2c-d). In addition, higher frequencies of Tfh cells were induced by DNA BG505 NLT than previously published Tfh frequencies for BG505.SOSIP immunizations in mice^17^.

We next characterized humoral responses induced in mice following DNA-encoded versus protein BG505.MD39 vaccination. While DNA-encoded BG505.MD39 induced stronger trimer (BG505.MD39)-specific binding antibody responses than protein BG505.MD39 at week 2 and week 5, binding titers at week 8 were similar (Fig. 2e). Strikingly, however, autologous Tier-2 neutralization against BG505.T332N using the TZM-bl assay was uniquely detected in 3 out of 10 mice immunized with DNA-encoded BG505.MD39, but not in naïve mice, mice immunized with pVAX1 plasmid backbone, or those immunized with protein BG505.MD39 two weeks post the third vaccination (Fig. 2f and Supplementary Table 1). While the neutralizing antibody titers (mean = 94) in the mouse responders were significantly lower than the average titers induced in rabbits (mean = 1000) by protein-based immunizations^46^ (particularly, neutralization titers of the two of the three responders were slightly above the limit of detection), four immunizations over a course of 40 weeks were used in the prior study. Non-specific neutralization of MLV was not observed in any of the post-immune sera in our assays.

### nAb responses increased with lengthened vaccination intervals

While we observed a striking phenotypic difference between DNA versus protein BG505.MD39 immunizations in terms of induction of nAbs, few responders in the study successfully developed nAb responses, and the ID_50_ titers (sera dilution that corresponds to 50% reduction in infection by pseudovirus) induced in the responders were low. Longer intervals between vaccinations have previously been demonstrated to induce superior antibody responses by allowing time for somatic hypermutation and affinity maturation^47,48^. We next explored if lengthening the interval between the second and third immunizations could increase the frequency of responders and magnitudes of responses.

**Fig. 3:**
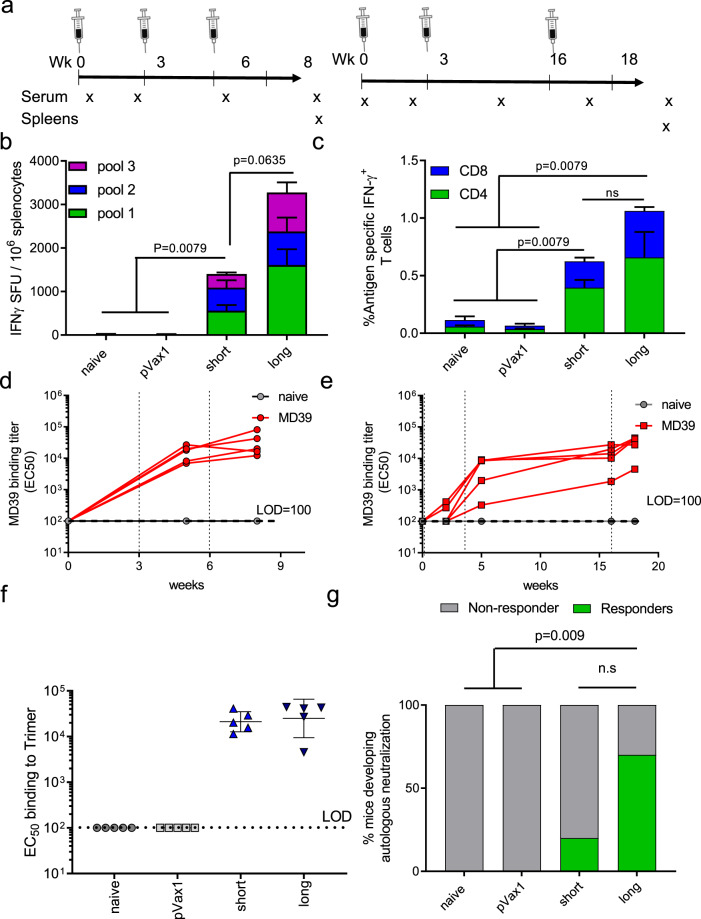
Prolonging the duration between the second and third immunizations increased frequencies of responders who developed autologous Tier-2 NAbs. Mice in this panel were immunized either with a short scheme (Wks 0, 3, 6) or a long scheme (Wks 0, 3, and 16) with 25 ug DNA used at each vaccination. The mice were euthanized 2 weeks post the final vaccination for cellular analyses. **a** Vaccination scheme used in this study. **b**, **c** Comparison of BG505 Env-specific cellular responses in naïve mice, mice immunized with pVAX1 plasmid backbone, or those immunized with DNA-encoded BG505.MD39 using either the short or the long scheme by IFNγ ELISpot assay (**b**) or intracellular cytokine staining (**c**). **d**, **e** Time course of trimer-specific binding antibodies in naïve mice or mice vaccinated with DNA-encoded BG505.MD39 using either the short scheme (**d**) or the long scheme (**e**) as determined by ELISA. **f** Comparison of trimer-specific binding antibodies at the final timepoint (2 weeks post the third vaccination) in mice immunized with DNA-encoded BG505.MD39 using either the short or the long scheme. **g** Frequencies of mice that developed autologous BG505.T332N neutralizing antibodies (ID_50_ titers greater than 1:45) in naïve mice, mice immunized with pVAX1, or mice immunized with DNA-encoded BG505.MD39 using either the short or the long scheme two weeks post the final vaccination. The dashed vertical line in **d** and **e** refers to the immunization timepoints. Two independent experiments were performed for each panel in the figure. *N* = 5 mice/group for **a**–**f**; *N* = 10 mice/group (**g**); each dot or line represents an animal. Error bar represents standard deviation. Center of the error bar represents the mean. Two-tailed Mann–Whitney Rank test used to compare groups; *p*-values were adjusted for multiple comparison for panels **b**, **c**. Two-sided Fisher Exact Tests were used to compare proportions of responders between long boost SynDNA group and naïve/ pVAX1/ short boost SynDNA groups in **g** with *p*-values adjusted for multiple comparisons. ns not significant. Source data are provided as a Source Data file.

BALB/c mice were immunized with 25ug of DNA-encoded MD39 at weeks 0, 3, 6 or weeks 0, 3, 16 (Fig. 3a). Mice immunized with the longer interval induced significantly higher antigen-specific T-cell responses as measured by IFN-γ ELIspot assays compared to mice receiving the shorter immunization schedule (Fig. 3b). In both cases, antigen-specific CD4^+^ and CD8^+^ T-cells were induced by DNA vaccinations (Fig. 3c). Interestingly, vaccination using the long interval scheme induced stronger polyfunctional CD4^+^ T-cell responses whereas vaccination with the short interval scheme induced stronger polyfunctional CD8^+^ T-cell responses as measured two weeks post last boost (Supplementary Fig. 5A-B). Both vaccine regimens induced strong trimer-specific binding antibodies with comparable titers two weeks post the final vaccination (Fig. 3d-f). It was observed that antibody responses did not contract significantly from week 5 (post 2^nd^ immunization) to week 16 (prior to 3rd immunization) (Fig. 3e). Importantly, in terms of nAb responses, it was observed that there was a higher frequency of responders (7 out of 10 mice) that developed autologous Tier-2 nAbs for the long vaccination scheme as compared to the short vaccination scheme (2 out of 10 mice). The long boost group also had a significantly higher proportion of responders than the naïve and pVAX1 groups (*p*-value = 0.009) (Fig. 3g). nAb ID_50_ titers in the responders were also found to be improved for mice that received the long immunization scheme (Supplementary Table 2). It may therefore be concluded that the potency and consistency of nAb responses induced by DNA-encoded NLTs may be further improved with optimized immunization regimens.

### Screening with engineered pseudoviruses mapped induced murine nAb responses to the Env C3/V5 epitope

**Fig. 4:**
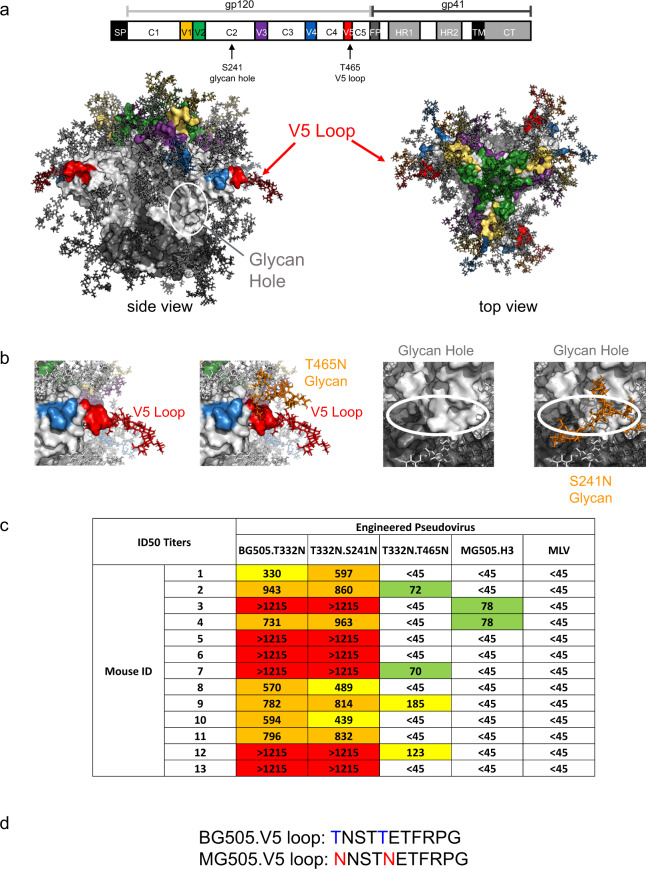
Screening with engineered BG505.T332N pseudoviruses with modified glycosylation sites mapped induced murine NAb to the V5 loop of Envelope. Mice in this panel were immunized with variations of DNA-encoded BG505.MD39 at Wks 0, 3, and 16 with 25ug DNA used at each vaccination and identified to possess potent BG505.T332N neutralizing activities 2 weeks post the final vaccination. **a**, **b** Rosetta modeling to demonstrate the locations of the engineered glycosylation sites (**a**) and the V5 loop (**b**) on the NLT. **c** Neutralization ID50 titers of heat-inactivated mice sera against listed pseudovirus isolates. **d** Sequence alignment of the V5 loops for BG505.W6M.ENV.C2 and MG505.W0M.ENV.H3.

We further interrogated nAbs induced in mice by determining the neutralization sensitivity to engineered pseudoviruses. BG505.T332N pseudovirus was used for mapping studies as the vaccine encoded stabilized BG505.MD39 also contains this corresponding glycan addition. Soluble BG505.SOSIP.664 protein could induce autologous Tier-2 neutralizing antibodies in rabbits, which were specific for the S241 glycan hole^49^ (Fig. 4a), and the V5 loop (C3/V5 epitope) in the case of rabbits and macaques^13,15,20,50–52^ and in SHIV-BG505 infected macaques^50,52^ (Fig. 4a, b). To determine whether the induced murine nAbs might be specific for these previously identified neutralizing epitopes, we engineered and titered two additional pseudoviruses containing point mutations BG505.T332N.S241N or BG505.T332N.T465N with a rationale that the introduced N-linked glycan may block binding and neutralization mediated by epitope-specific antibodies^53^. We performed screening using post-immune mice sera that have previously been demonstrated to possess potent neutralizing activities against the autologous BG505.T332N virus. We observed that while the murine nAbs could neutralize the S241N isolate almost as effectively, nAbs were far less potent in their activities against the T465N isolate, suggesting that the induced nAbs likely to target the V5 loop but not the S241 glycan hole (Fig. 4c). Expectedly, induced murine nAbs also failed to neutralize the maternal MG505.H3 isolate, which differed in its sequence of the V5 loop by two residues (T to N changes) as compared to the BG505 isolate^19,54^ (Fig. 4d). We did not observe neutralization of any of the pseudovirus isolates in the global panel^55^.

### Single Cell RNA sequencing on antigen-specific B-cell recovered monoclonal murine nAb that targeted the V5 loop

**Fig. 5:**
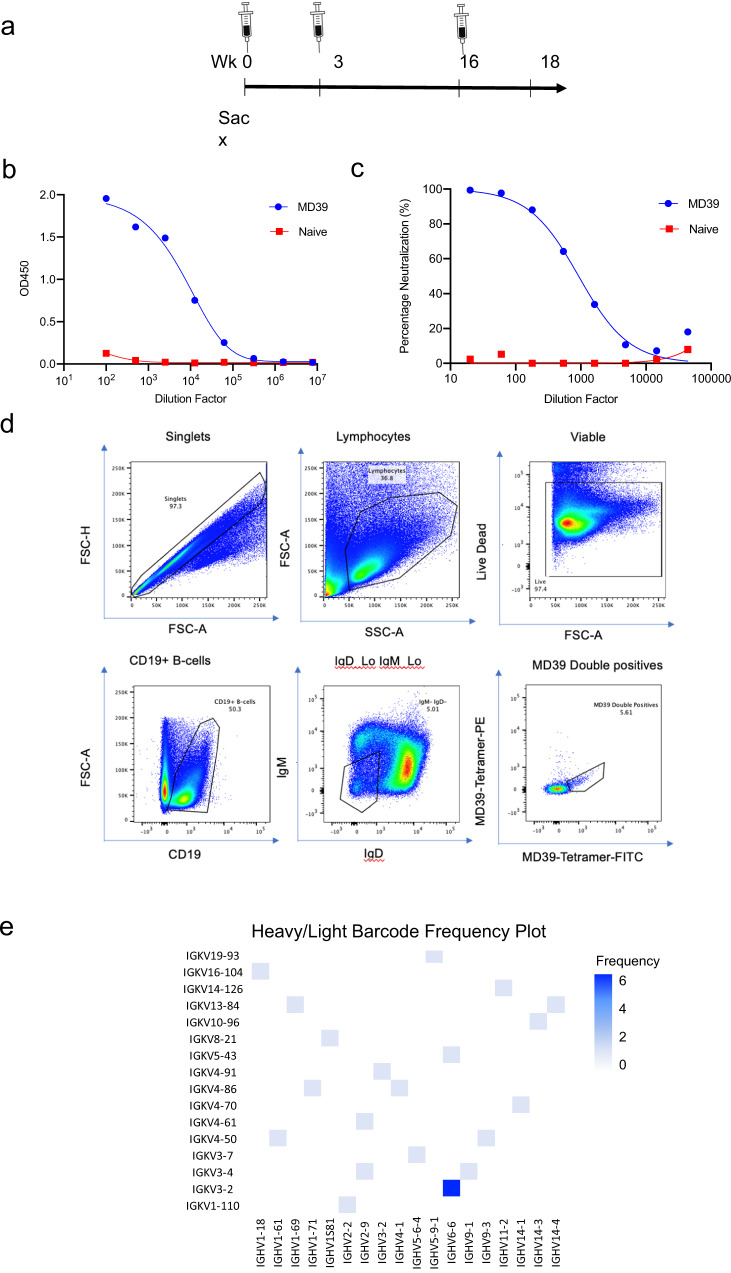
Isolation of trimer-specific monoclonal antibodies from an animal (272.8) with potent neutralization activity against BG505.T332N post vaccinations with DNA-encoded BG505.MD39. **a** Vaccination and tissue collection scheme for 272.8. **b** Trimer-specific binding antibody responses in 272.8 2 weeks post the final vaccination. **c** Autologous BG505.T332N neutralizing activity induced in 272.8 2 weeks post the final vaccination. **d** Gating strategy used to isolate antigen-specific CD19 + IgD− IgM− BG505.MD39-Tetramer-FITC + BG505.MD39-Tetramer-PE + B-cells from spleen and lymph nodes of 272.8 2 weeks post the final vaccination. **e** Characteristics of isolated murine monoclonal antibodies in terms of germline VH and VL gene usage.

To further validate our findings and characterize induced murine nAb responses, we immunized a new group of mice and attempted to recover monoclonal antibodies from one animal (272.8) that developed nAb responses against the BG505.T332N isolate following DNA-encoded BG505.MD39 immunization. The animal was immunized according to the long vaccination regimen (Wks 0, 3, 16) (Fig. 5a), and was euthanized two weeks post the final vaccination for tissue (spleen and draining lymph nodes) collection. At the final timepoint, potent trimer-binding antibodies were induced in 272.8 (Fig. 5b). The serum of the animal also demonstrated strong neutralization activity against the BG505.T332N isolate, conferring almost complete neutralization of the virus at a low dilution (Fig. 5c). Collected spleen and lymph nodes were homogenized into a single-cell suspension and stained with relevant surface antibodies and fluorophore-conjugated streptavidin-scaffolded avi-tagged BG505.MD39 tetramers as previously described^8,56^. Viable antigen-specific CD19^+^ IgM^−^IgD^−^ MD39_Tetramer_FITC^+^ MD39_Tetramer_PE^+^ B cells were subsequently sorted out in bulk with a FACS ARIA II instrument (Fig. 5d) for single-cell RNA sequencing. By using the 10× genomics platform, it was possible to retrieve paired heavy and light chain sequences for each B cell that had been bulk sorted^57^. Following cDNA prep, enrichment of VH and VL DNA fragments, and next-generation high-throughput sequencing, 25 unique clones were identified (Supplementary Fig. 6A-B). Eighteen unique germline VH genes were identified among the 25 clones as well as 16 unique germline VK genes with a high frequency of one IGHV/IGKV pairing (IGHV6-6/IGKV3-2) (Fig. 5e and Table 1). While we recovered many unique cDNA reads that corresponded to the IGHV6-6/IGKV3-2 antibody pair, the most prevalent cDNA read corresponded to a single clone was from an IGHV3-2/IGKV4-91 antibody pair, clone 3 (C03) (Table 1). The 25 unique clones displayed variable HCDR3 lengths from 6 amino acids to 17 amino acids and variable HCDR3 sequences; however, the 6 common IGHV6-6 clones were consistent in both HCDR3 lengths and had HCDR3 sequence convergence (Supplementary Fig. 6B).

**Table 1:** Detailed characterization of isolated murine BG505.MD39-specific clones in terms of VH, VJ, VD, VK, VK, VJ gene usage, HCDR3 and LCDR3 lengths.

Antibody clone	HV gene	HD gene	HJ gene	CDRH3 length	HV %identity	KV gene	KJ gene	CDRK3 length	KV %identity	Barcode percentage
C3	3-2*02	2-4*01	4*01	12	100.00	4-91*01	2*01	9	100.00	12.5
C4	14-1*02	1-1*01	2*01	9	100.00	4-70*01	4*01	8	100.00	9.375
C5	6-6*02	1-2*01	4*01	13	95.92	3-2*01	2*01	9	96.91	9.375
C6	6-6*02	1-2*01	4*01	13	95.92	3-2*01	2*01	9	96.91	6.25
C10	5-6-4*01	2-2*01	2*01	17	88.54	3-7*01	1*01 1*02	9	91.41	3.125
C11	6-6*02	1-2*01	2*01	10	98.98	5-43*01	2*01	10	100.00	3.125
C13	11-2*02	2-3*01	1*01	12	100.00	14-126*01	5*01	9	100.00	3.125
C16	14-4*02	1-2*01	2*01	13	100.00	13-84*01	1*01	8	100.00	3.125
C18	1-61*01	2-4*01	3*01	12	96.53	4-50*01	5*01	9	100.00	3.125
C19	2-9*02	3-1*01	4*01	9	100.00	4-61*01 3-4*01	5*01 1*01	9	100.00	3.125
C20	1-69*02 1S126*01	—	2*01	8	94.44	13-84*01	5*01	9	100.00	3.125
C22	6-6*02	1-2*01	4*01	13	92.86	3-2*01	2*01	9	96.56	3.125
C23	4-1*02	2-3*01	3*01	11	95.83	4-86*01	5*01	9	97.46	3.125
C24	6-6*02	1-2*01	4*01	13	92.86	3-2*01	2*01	9	98.28	3.125
C25	9-1*02	2-4*01	4*01	12	100.00	3-4*01	2*01	9	100.00	3.125
C26	14-3*02	1-2*01	2*01	9	100.00	10-96*01	2*01	9	100.00	3.125
C27	1-62-2*01 1-71*01	2-1*01	3*01	14	99.31	4-86*01	1*01	9	100.00	3.125
C28	2-2*02	2-10*01	3*01	13	100.00	1-110*01	2*01	9	100.00	3.125
C30	1-18*01 1-22*01	3-1*01	4*01	11	96.53	16-104*01	2*01	9	99.64	3.125
C32	9-3*02 9-3*03	1-1*01	3*01	13	100.00	4-50*01	5*01	9	100.00	3.125
C33	6-6*02	1-2*01	4*01	13	97.28	3-2*01	2*01	9	100.00	3.125
C34	5-9-1*01	2-10*02	2*01	10	100.00	19-93*01	2*01	8	100.00	3.125
C35	1S81*02	—	2*01	6	96.53	8-21*01	5*01	8	100.00	3.125
C37	6-6*02	1-2*01	4*01	13	95.92	3-2*01	2*01	9	97.94	3.125

**Fig. 6:**
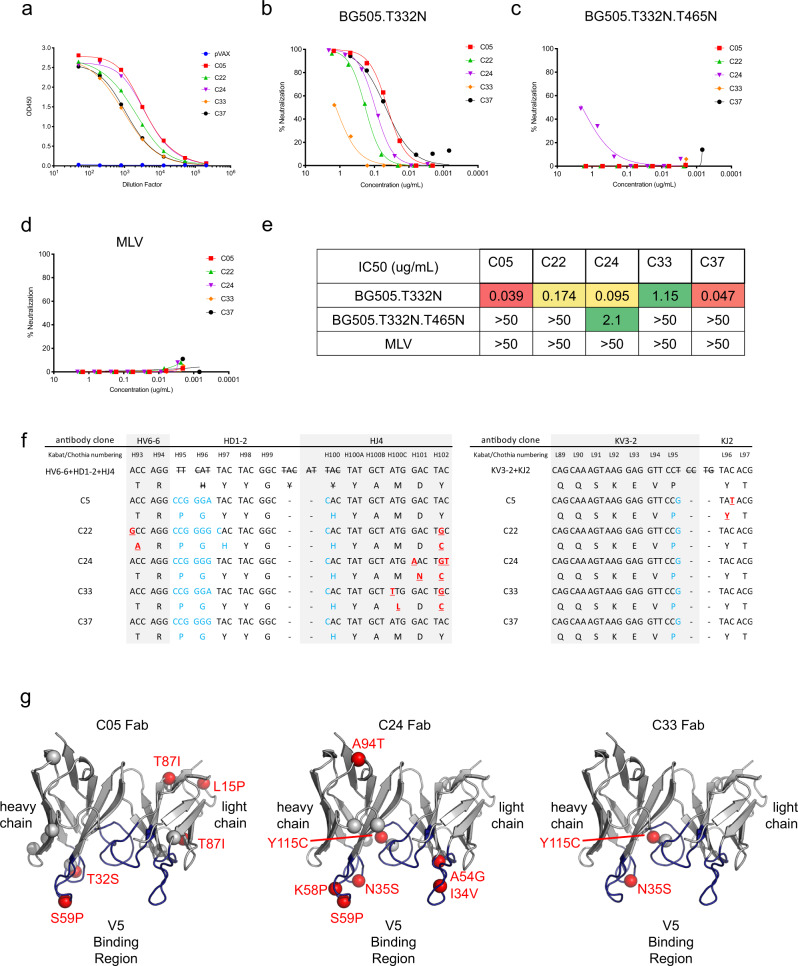
Isolated trimer-specific murine MAbs neutralized BG505.T332N pseudovirus in a V5-dependent fashion. **a** Trimer-specific binding of 5 isolated murine MAbs and PGT128 in Expi293F transfection supernatant. **b**–**d** Neutralization of BG505.T332N (**b**), BG505.T332N.T465N (**c**), and MLV (**d**) pseudoviruses with varying concentrations of murine trimer-specific MAbs or PGT128. **e** Determined IC50 values in ug/mL of trimer-specific murine MAbs for BG505.T332N, BG505.T332N.T465N, or MLV. **f** Amino acid alignments of the HCDR3 and LCDR3 regions of the identified BG505.MD39-specific murine antibody clones as compared to the corresponding germline sequences. **g** Predicted Rosetta structures of the Fabs of clones C05, C24, and C33, positions mutated from the germline sequences are displayed in red.

Next, we generated a 2 plasmid-system DNA assembly of the enriched clones. One plasmid consists of each variable heavy fragment upstream of human IgG1 Fc/CH1 and the other plasmid contains each variable light fragment upstream of the constant domain of the human Kappa light chain. These were dually expressed using small-scale transfections of Expi293F cells and the supernatants screened with MD39 trimer-binding ELISA (Fig. 6a). 5 out of 25 clones (C05, C22, C24, C33, and C37) demonstrated appreciable binding to both MD39 and gp120-foldon with similar affinities in terms of EC50 (Supplementary Fig. 7A–C) and their in vitro expression was characterized by western blot (Supplementary Fig. 7D). Antibody concentrations of the supernatants were calculated by standard ELISA binding curves for the five clones using purified IgG (Supplementary Fig. 7E). We next evaluated the neutralizing activities of the selected clones. All clones demonstrated appreciable neutralizing activities against BG505.T332N to varying degrees (Fig. 6b and Supplementary Fig. 7F), the potency of which were observed to be similar to rhesus nAbs 1B1 and 1B6 (IC_50_ values of 0.03 and 0.06 ug/mL, respectively)^52^. Simultaneously, none of the clones could completely neutralize BG505.T332N.T465N (Fig. 6c), which remained sensitive to neutralization by V3 N332-directed bNAb PGT128 (Supplementary Fig. 7G)^58^, demonstrating V5-specificities of the isolated clones. In comparison, PGT128 neutralized BG505.T332N.T465N as efficiently as it did for BG505.T332N (Supplementary Fig. 7G). One clone, C24, demonstrated modest neutralization of the BG505.T332N.T465N which could indicate a slightly modified paratope from the other clones. None of the isolated murine mAb clones non-specifically neutralized control pseudovirus MLV (Fig. 6d). Overall, the five isolated clones displayed different potencies in the neutralization of autologous BG505.T332N pseudovirus, with C05 and C37 being the most potent clones, with IC_50_ values of 37 and 47 ng/mL, respectively (Fig. 6e). The five functional clones were of the same common antibody lineage IGHV6-6/IGKV3-2 with similar HCDR3 loops (Fig. 6f). The kappa light chains showed few variances between clones ranging from 2 amino acids to 11 amino acids per variable gene. The heavy chains displayed more variation with 7 amino acids to 15 amino acids per gene (Supplementary Fig. 8A). Clones C05, C24, and C33 were structurally modeled and compared due to the differences in IC_50_ values and neutralization of BG505.T332N.T465N pseudovirus. A previously crystalized murine IGHV6-6/IGKV3-2 paired antibody was used as a template^59^ and the somatic mutations and variable loops were modeled with Rosetta (Fig. 6g). Some mutations were common among multiple clones (Fig. 6g, red spheres) such as the N35S mutation found on the HCDR1 loop which was mutated in four out of the five functional antibodies while many framework mutations were unique to each clone (Fig. 6g, gray spheres). Clone C33 had the fewest mutations away from the germline (only 3 mutations in total) and the weakest neutralization against BG505.T332N pseudovirus. C24 has a greater number of CDR loop mutations compared to C05 which could account for the differences in the neutralization of the BG505.T332N.T465N pseudovirus (Supplementary Fig. 8B). To further investigate the epitope specificity of C24 antibody in comparison with other isolated antibodies, we performed competition ELISA and determined whether each of the following non-biotinylated antibodies (C05, C22, C24, C33, C37, and PGT128) could block the binding of biotinylated C24 to MD39 (Supplementary Fig. 9A). We observed that while C05, C22, and C37 could outcompete the binding of biotinylated C24 to MD39, C33, and PGT128 were unable to do so (Supplementary Fig. 9A-B), suggesting C05, C22, C24, and C37 may bind to MD39 in a similar fashion. While the result may suggest C33 approaching MD39 from a slightly different angle as compared to C24, it may also be due to lack of competition by C33 due to intrinsic lower binding affinity to MD39 (Fig. 6e). We performed an additional competition ELISA to determine whether non-biotinylated antibodies (C05, C22, C24, C33, C37, and PGT128) could block the binding of biotinylated C33 to MD39, observing that C05, C22, C24, and C37 could outcompete binding of biotinylated C33 to MD39, while PGT128 was unable to do so (Supplementary Fig. 9C-D). Additionally, we performed SPR kinetic analysis to assess the binding affinity of C24 and C33 to GP120 (to preserve 1:1 binding ratio) confirmed lower affinity of C33 with K_D_ at ~682 nM as compared to 23 nM for C24 (Supplementary Fig. 9E-F). Taken together, the results suggest C05, C22, C24, C33, and C37 likely approach target epitope similarly with C33 having lower binding affinity than the rest of the antibodies.

### Cryo-EM characterization of Fab of murine nAb bound to MD39 provided a structural basis for C3/V5-directed neutralization

**Fig. 7:**
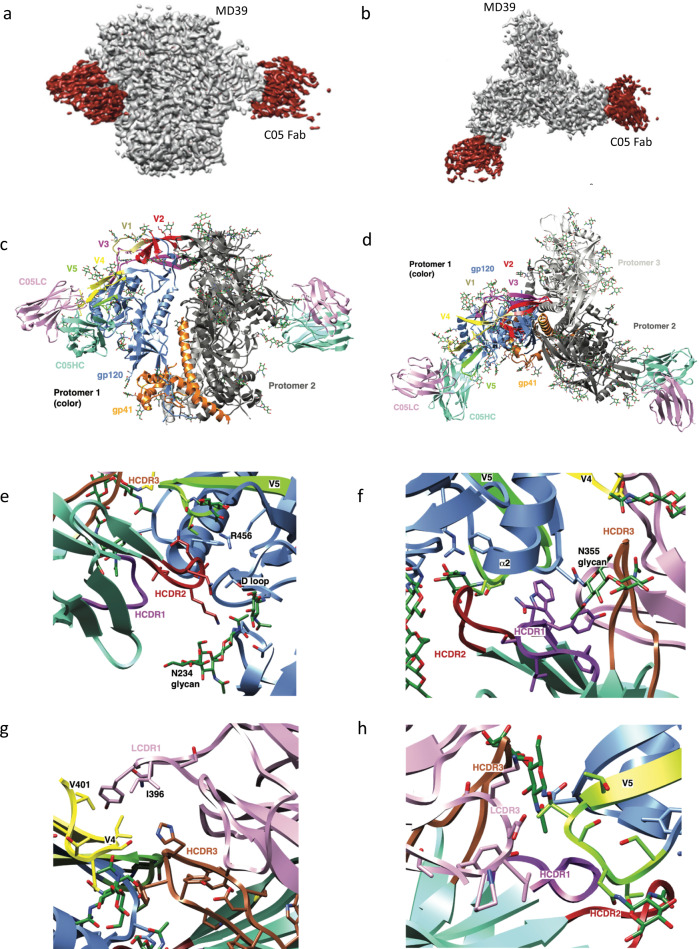
Cryo-EM structure of MD39 in complex with C05 Fab. **a**, **b** Electron density map of MD39 C05 Fab complex viewed from the side (**a**) and from the top (**b**); MD39 is shown in gray; C05 Fab is shown in dark red. **c** Side view and **d** top view of the modelled structure. Env Protomer 1 is displayed in color, protomer 2 in dim gray, and protomer 3 in light gray. Env protomer 1 gp120 is displayed in cornflower blue, gp41 in orange, and N-linked glycans in forest green. Env variable region 1 (V1) is displayed in khaki, V2 in red, V3 in magenta, V4 in yellow and V5 in chartreuse. C05 Fab variable domains are displayed in plum (Fv light chain; C05LC) and in aquamarine (Fv heavy chain; C05HC). C05 recognizes an epitope that comprises C3 and V5, but also part of V4. **e** HCDR2 (firebrick) engages the Env D-loop, Env N234 glycan, and R456. **f** HCDR1 (purple) engages the base of Env helix α2 as well as Env N355 glycan. N355 glycan is involved in HCDR3 (sienna) engagement as well. **g** HCDR3 (sienna) further engages the N-terminal part of V4 (yellow). **h** LCDR3 (plum) recognizes V5 (chartreuse).

To confirm our findings that the C3/V5 epitope is the precise target of the monoclonal nAbs, we employed Cryo-EM electron microscopy. C05, which is the most potent neutralizer of BG505 pseudovirus (Fig. 6e), was chosen for structural characterization. A complex of a BG505.MD39 version with C05 Fab was formed and we obtained a density map at 3.8 Å overall resolution (Supplementary Fig. 10, Supplementary Table 3). In our structure (Fig. 7a–d), only two of the three exposed epitopes are occupied by C05 Fab, despite extended incubation time with a large excess of C05 Fab. Our structure confirms that indeed C05 Fab variable region recognizes a conformational epitope comprising C3/V5 Env regions. The solvent-excluded epitope/paratope area between C05 and Env amounts to 479Å^2^. C05 HCDR2 recognizes a pocket that comprises the D-loop (E275-N283), V5 (S460-R469), the N234 glycan, and R456 (Fig. 7e). The latter (R456) partakes in CD4 engagement and engagement of R456 by C05 HCDR2 suggests that occlusion of CD4 binding may be part of its neutralization mechanism. HCDR1 further stabilizes C05 binding by interacting with the base of helix α2 and its associated N355 glycan bridging the paratope gap toward HCDR3 (Fig. 7f). HCDR3 makes extensive contact with the N-terminal part of V4 thereby facilitating order in this region (N386-V401; Fig. 7g). Interestingly, W395-V401 folds back on itself to display I396 and V401 for stabilizing interaction with LCDR1 (Fig. 7g). LCDR3 recognizes V5 and, along with HCDR2 dictates order in V5 (Fig. 7h).

Next, we sought to compare structures of the murine C3/V5 neutralizing antibodies to structures of C3/V5 targeting antibodies from other species. The C3/V5 epitope has been primarily mapped by negative-stain electron microscopy of the post-immune polyclonal sera and has been identified in NLT-immunized rabbits^60^, NLT-immunized Guinea Pigs^61^, NLT-immunized macaques^13,50–52^ and SHIV-infected macaques^50,52^. The antibodies from these studies engage the C3/V5 epitope from various angles of approach. The murine antibodies seem to engage the epitope most similarly to antibodies in a polyclonal EM structure from a NLT vaccinated rhesus macaque with high titers of neutralizing antibodies, which was fully protected after 12 SHIV challenges^50^ (Supplementary Fig. 11). While the polyclonal EM method is incredibly powerful, it does not map the functionality of each clone to the structure. Here, we have the mapping of C3/V5-targeting antibody structure and function, allowing us to identify the C3/V5 engagement geometry that is consistent with neutralization.

In summary, using current molecular biology and genomics techniques, we identified 5 related and novel murine C3/V5-directed nAbs and validated that mice can mount epitope-specific nAb against an autologous Tier-2 isolate following DNA-encoded NLT immunization.

## Discussion

While iterative vaccine studies are critical to the development of an efficacious HIV vaccine, a significant bottleneck for studying NLT-induced neutralizing immune responses has been the reliance on large animal models. To compound the problem of increased cost and lengthy experiment times associated with larger animals, recombinant protein immunogens required in most NLT studies to date necessitate costly and arduous expression and purification approaches. Here, we present a potential breakthrough in this long-standing bottleneck in HIV research and demonstrate a simple, rapid and cost-effective strategy to screen for antigens that elicit neutralizing antibodies, which involves immunization of BALB/c mice with DNA-encoded NLTs to enable rapid immunological read-outs.

A long-time collective effort in HIV vaccine research has been focused on producing immunogens with proper trimer folding and concomitant neutralizing antibody responses. Given DNA NLTs were produced in vivo, it was important to confirm the assembly of our NLT immunogen in vivo. Previous studies involving DNA vaccination of NLTs did not directly measure antigen assembly in the animals and did not demonstrate that DNA vaccinations alone could induce Tier-2 autologous neutralizing antibodies^36^. In this study, we sought to provide a more direct assessment of in vivo assembly of conformationally complex HIV NLT antigens. First, we performed Native PAGE electrophoresis on immunized muscle homogenates to obtain protein separation of material being produced in vivo roughly by molecular weight, observing in vivo produced materials migrated in a similar fashion compared to recombinantly produced BG505.MD39. The slight difference in migration patterns between the two was likely due to glycosylation differences by different host cell types, a subject of future study. Second, we performed in vivo antigenic profiling of NLTs for what we believe is the first time using both immunofluorescence and ELISA techniques. We observed that DNA/EP delivery of BG505.MD39 results in direct in vivo production of cleaved trimeric antigens, which retain important conformational epitopes (e.g. V2-apex site, fusion peptide) that are unique to NLT Env immunogens.

The neutralizing specificities elicited from the murine antibody repertoire against NLTs relative to other animal models have not been well described. The serological and structural data generated by BG505.MD39 immunizations show for the first time that immune systems of BALB/c mice can target the C3/V5 neutralizing epitope which is a broadly targeted epitope capable of mediating protection from viral challenges in other large animal species^13,15,20,50–52^. Interestingly, in a recent bolus vs extended dose study of BG505 NLTs in rhesus macaques, the extended dose group uniquely targeted C3/V5 and these animals had much higher neutralizing titers^51^. C3/V5-targeting nAbs are the dominant neutralizing response in protected macaques^52^. In electron microscopy-based mapping studies using polyclonal sera the C3/V5 epitope can be engaged from several different angles of approach^50^. Here, we use this in vivo NLT immunization model to generate a collection of mAb that allows us to define the angle of approach that is used to neutralize via the C3/V5 epitope by determining a cryo-EM structure of bound monoclonal neutralizing abs. To our knowledge, the atomic resolution structure reported here is the first high-resolution structure of a Tier-2 nAb binding to C3/V5. The extensive interaction between C05 HCDR2/HCDR3 with Env V4/V5 provided a structural basis for neutralization. This cross-species epitope may be a relevant target of vaccine-induced human immunity.

By comparing protein to DNA immunizations of BG505.MD39 in mice at the equivalent dose, we observed different immunity. This was unexcepted as both routes ultimately should be presenting the same structure and distinct epitopes to the immune system. While both protein and DNA vaccination-induced trimer-specific binding antibodies, DNA but not protein vaccination-induced CD4^+^ and CD8^+^ T-cell responses as well as nAb responses. Differences in the mechanisms with which antigens traffic and present for protein versus DNA-encoded immunogens may underlie differential induction of immune phenotypes by these two routes of vaccination. Protein antigens are supplied as a depot of soluble antigens and may rely on bulk flow in the lymphatic system for antigen trafficking^62^. In contrast, it has been shown that DNA vaccination may either result in direct transfection of antigen-presenting cells (APCs) to express the encoded antigen or may result in transfection of myocytes which can then transfer the expressed antigens to infiltrating APCs through the formation of apoptotic bodies^63,64^. Such differences in antigen-presentation may partially explain the unique induction of CD8^+^ T-cell responses as explored in prior work and may also underlie differences in the epitope specificities of the induced antibodies. More study of such platform-specific mechanisms will likely be informative.

There are unanswered questions that remain with this approach that should be explored in future work. The glycosylation profiles of in vivo produced trimers, for instance, may be further interrogated with more sophisticated purification techniques (tag-based purification or FPLC), protein digestion, and downstream mass spectrometry analyses of the liberated glycopeptides to establish a correlation between the glycosylation profile and specificities of induced antibodies^65^. Consistency and potency of the nAbs induced by DNA-encoded BG505.MD39 immunizations may be further improved with optimization in the dosing intervals. We observed here that lengthening the third dose from 6 weeks to 16 weeks improved neutralizing responder rate. This observation is consistent with prior work that delayed boost may enhance secondary antibody responses by allowing more time for somatic hypermutations and affinity maturation of germinal center B cells^66^. Clearly, translation of these findings in larger animal species would be of interest for clinical development. This DNA-EP platform has recently shown >98% antibody seroconversion rates in a clinical HIV vaccine study^67^ and is being employed to administer a SARS-CoV-2 vaccine^23,68,69^. In addition, the neutralizing immunity directed at C3/V5 elicited by DNA immunization of BG505.MD39 is considerably specific, as we did not observe neutralization breadth across other viruses in the global panel^70^. Therefore, it will be important to consider strategies that may broaden neutralization mediated by the C3/V5 epitope.

Despite these challenges, DNA vaccination still remains an important strategy for in vivo delivery of NLTs, considering the cost-effectiveness of DNA production, the high thermal stability of DNA plasmids^71,72^, the ability of DNA to potentiate in vivo expression of conformationally complex immunogens driven from larger cassettes to induce unique and relevant immune phenotypes^31,73^. The present study opens the door for exciting new studies aiming to deliver complex NLTs, such as cocktails or germline-targeting immunogens, to rapidly execute pre-clinical NLT studies and to translate NLT immunogens to the clinic.

## Methods

### DNA design and plasmid synthesis

Amino acid sequence for BG505_MD39 based stabilized trimers was obtained from previously published sequence^32^. Control plasmid encoding the gp120_foldon was designed as previously described^5^. The full-length unmodified BG505 gp160 sequence was also made using the sequence from GenBank (accession number DQ208458). These sequences were then RNA and codon-optimized as well as optimizing for GC content and secondary structure. Additionally, an optimized IgE leader sequence was added to the N terminus of the protein to provide efficient processing and secretion. Variable heavy and light chain sequences of isolated murine antibody clones were combined downstream with human IgG1 Fc or Kappa constant light chain. All plasmids were synthesized and cloned (GenScript) into our modified pVAX1 backbone (Inovio Pharmaceuticals).

The full-length BG505 plasmid was used to make point mutations at T332N, T332N S241N, and T332N T456N for pseudotype virus production. Plasmids expressing MG505.H3 Env, HIV backbone ΔEnv (pSG3), and murine leukemia virus control Envelope were obtained from NIH AIDS Reagents Program.

### Cell lines, transfection, and recombinant antibody purification

HEK 293 T cells (ATCC) and TZM-bl cells (NIH AIDS Reagents Program) were maintained in DMEM (ThermoFisher) supplemented with 10% heat-inactivated fetal bovine serum (Atlas Biologicals). Expi293F cells (ThermoFisher) were maintained in Expi293 expression medium (ThermoFisher). All cell lines were mycoplasma negative and tested on a regular basis. To produce recombinant HIV monoclonal antibodies for small-scale assays, Expi293F cells were transfected following the manufacturer’s protocol for Expifectamine. Transfection enhancers were added 18 h after transfection and supernatants were harvested 6 days later. For large-scale antibody production, Expi293F cells were transfected using PEI, and supernatants were harvested 6 days later. Protein A agarose was used following the manufacturer’s protocol to purify the IgG. Purity was confirmed with commassie staining of SDS-page gels and concentration was determined using a nanodrop. Supernatant IgG expression was quantified using a standard binding curve from the large-scale purified antibodies in an ELISA.

Pseudotype viruses were produced as previously described^74^. Briefly, pseudotyped viruses were produced using HEK 293 T cells transfected with 4 ug of a plasmid expressing the Env of interest and 8 ug of a plasmid expressing the HIV-1 backbone Δ Env (pSG3ΔEnv − NIH AIDS Reagents) using GeneJammer (Aglient). Forty-eight hours after transfection, cell supernatant was harvested, filtered through a 45 µm filter, aliquoted, and stored at −80 °C.

### Production of trimer

BG505_MD39-based trimers were expressed in FreeStyle 293 F Cells (Invitrogen) and are derived from a low-passage Master Cell Bank and certified mycoplasma free. The trimer-containing supernatants were obtained by centrifuging (4000 × *g*, 25 mins) and filtering (0.2 um Nalgene Rapid-Flow Filter) the 293 F cultures. Trimers were purified from supernatants by lectin purification using lectin beads (Vector Laboratories 7.5 ml beads/1 L culture) and lectin elution buffer (1 M Methyl alpha-D-mannopyranoside). The elution was dialyzed overnight into PBS. The trimers were then purified over a size-exclusion chromatography column (GE S200 Increase) in PBS. The molecular weight and homogeneity of the trimers were confirmed by protein conjugated analysis from ASTRA with data collected from a size-exclusion chromatography-multi-angle light scattering (SEC-MALS) experiment run in PBS using a GE S6 Increase column followed by DAWN HELEOS II and Optilab T-rEX detectors. The trimers were aliquoted at 1 mg/ml and flash frozen in thin-walled PCR tubes prior to use.

### Negative stain EM of purified trimers

SEC purified MD39 trimers were further dialyzed into Tris‐buffered saline (TBS). A total of 3 µL of purified proteins was adsorbed onto glow discharged carbon‐coated Cu400 EM grids. The grids were then stained with 3 µL of 2% uranyl acetate, blotted, and stained again with 3 µL of the stain followed by a final blot. Image collection and data processing were performed on a FEI Tecnai T12 microscope equipped with Oneview Gatan camera at 90 450× magnification at the camera and a pixel size of 1.66 Å.

### Immunization of Mice

All mice were housed in compliance with the NIH and Wistar’s Institutional Animal Care and Use Committee guidelines under IACUC protocol 201214. To test for immunogenicity, 6–8 week old BALB/c mice were immunized with 25 ug of plasmid followed by in vivo electroporation using the CELLECTA® 3 P adaptive constant current electroporation device (Inovio Pharmaceuticals) or immunized with 25 ug of protein co-formulated with Sigma Adjuvant System (RIBI) subcutaneously over the flanks. Mice were immunized at either 0, 3, 6 weeks or 0, 3, 16 weeks and sacrificed two weeks after final immunization to determine vaccine-induced immune responses. Sera were collected at the indicated timepoint through the submandibular vein for assessment of humoral responses. Spleens, iliac, popliteal, and inguinal lymph nodes were collected from euthanized animals at specified timepoints for analyses of cellular responses. For NATIVE PAGE and IFA analyses of in vivo produced trimers, BALB/c mice received intramuscular injection of 100 ug DNA plasmid encoding either BG505.gp120.Foldon or MD39 co-formulated with 12U hyaluronidase (Sigma–Aldrich) in the tibialis anterior muscle, followed by IM-EP with the Cellectra 3 P device. 4 d.p.i (for NATIVE PAGE analysis) or 7 d.p.i (IFA), mice were euthanized for collection of the injected tibialis anterior muscle.

### Mouse muscle staining of in vivo produced antigens

For muscle staining, 7 days after BALB/c mice were immunized with 100 µg DNA plasmid co‐formulated with 12 U hyaluronidase, the tibialis anterior muscles of the mice were harvested and preserved in 4% PFA/PBS for 2 h at room temperature and then stored overnight in 70% EtOH/H_2_O at 4 °C. The tissues were then serially dehydrated and blocked in 3% BSA/PBS for 1 h at room temperature, followed by overnight staining with 6 µg/mL PGT145 or 3074 (NIH AIDS Reagent). The sections were then washed and stained with anti‐human Alexa Fluor 488 antibody (Invitrogen), counterstained with 0.5 µg/mL DAPI (Sigma–Aldrich), and imaged with a Leica SP5 confocal microscope.

### ACTIVE—Antigen Conformation Tracing in vivo by ELISA

For Antigen Conformation Tracing in vivo by ELISA, 5 days after BALB/c mice were immunized with 100 µg DNA plasmid co‐formulated with 12 U hyaluronidase, the tibialis anterior muscles of the mice were harvested and homogenized in T‐PER extraction buffer (Thermo Fisher Scientific) and protease inhibitor (Roche). Muscle homogenates were subsequently concentrated 20× with Amicon Ultra 0.5 mL centrifugation kits with 3kDA cutoffs (Millipore Sigma) and protein concentrations were quantified with BCA assays (Thermo Fisher Scientific). Elisa plates were coated with 2 ug/ml of recombinant PGT128 Fab fragments overnight in PBS. After washing, plates were blocked with 5% skim milk in PBS with 1% newborn calf serum (NBS) and 0.2% Tween for 1 h at RT. Total protein concentration of muscle lysate for in vivo produced NLT and gp120-foldon was normalized and serial dilutions were added to the plate. Recombinant NLT and gp120-foldon were added as positive control standards. Plates were incubated at RT for 2 h. Plates were washed, and antibody of Interest was added at 10 ug/ml except for PGT145 which was added at 50 ug/ml, for 1 h at 37 °C. Plates were washed and probed with Goat Anti-Human IgG Fc Fragment HRP conjugated (Bethyl Laboratories Inc). Plates were developed for 5 min with 1-step ultra TMB (ThermoFisher) and stopped with 1 N H_2_SO_4_. Absorbance at an optical density (OD) of 450 nm and 570 nm was measured on a Synergy2 plate reader (BioTek Instrument). The background 570 nm OD was subtracted from the 450 nm reading. The data was analyzed and fitted using Graph Pad Prism 9.0.

### NATIVE PAGE analysis of in vivo produced MD39

Tibialis anterior muscles of immunized animals were harvested and homogenized in T‐PER extraction buffer (Thermo Fisher Scientific) and protease inhibitor (Roche). Muscle homogenates were subsequently concentrated 20× with Amicon Ultra 0.5 mL centrifugation kits with 3kDA cutoffs (Milipore Sigma) and protein concentrations were quantified with BCA assays (Thermo Fisher Scientific).

For the Coomassie staining, varying amounts of MD39 or BG505 gp120 (NIH AIDS Reagent) protein standards were co-incubated with 1× NATIVE sample running buffer and loaded onto 3–12% Bis-Tris NATIVE PAGE gels (Invitrogen) along with Native Marks. After electrophoresis, the gel was stained with Coomassie Blue R-250 (BioRad) at room temperature for 30 min, followed by de-staining with dH_2_O.

For the western analysis, 50 µg muscle homogenates or protein standards were loaded onto 3–12% Bis-Tris NATIVE PAGE gel for electrophoresis. Proteins were subsequently transferred to PVDF membrane from the gels and stained overnight at 4 °C with 3 µg mL^–1^ 2G12 antibody (NIH AIDS Reagent) in Odyssey Blocking Buffer/PBS/0.1% Tween (LI‐COR Biosciences), and 1:10,000 IRDye 800CW goat anti‐human IgG (LI‐COR Biosciences) in Odyssey Blocking Buffer/0.1% Tween/0.1% SDS at room temperature for 1 h, and then scanned with LI‐COR Odyssey CLx.

### ELISA

#### Serological trimer-binding ELISA

Binding titers to trimer were determined by coating plates with 2ug/ml of recombinant PGT128 antibody overnight in PBS. After washing, plates were blocked with 5% skim milk in PBS with 1% newborn calf serum (NBS) and 0.2% Tween for 1 h at RT. Recombinant trimer was added at 4 ug/ml for 2 h at RT. Serum was serially diluted, added to plates, and incubated at 37 °C for 1 h. Antigen and species-specific IgG was then detected across absorbed secondary anti-mouse HRP antibody (Bethyl Laboratories Inc). Plates were developed for 5 min with 1-step ultra TMB (ThermoFisher) and stopped with 1 N H_2_SO_4_. Absorbance at an optical density (OD) of 450 nm and 570 nm was measured on a Synergy2 plate reader (BioTek Instrument). The background 570 nm OD was subtracted from the 450 nm reading.

#### Recombinant antibody binding ELISA

Antibody affinity to BG505.MD39 trimer was determined by coating plates with 1 ug/ml goat anti-his6X antibody in 1×PBS for 3 h at RT. After washing with 1×PBS containing 0.05% Tween, plates were blocked overnight with 1×PBS containing 0.1% Tween and 5% skim milk. Plates were washed and his-tagged BG505.MD39 was incubated at 1 ug/ml for 1 h at RT. Plates were washed and recombinant antibody was serially diluted, added to plates, and incubated for 1 h at RT. After washing the plates, goat anti-human IgG Fc (Bethyl Laboratories Inc) at a dilution of 1:10,000 was incubated for 1 h at RT. Plates were washed and developed for 5 min with 1-step ultra TMB (ThermoFisher) and stopped with 1 N H_2_SO_4_. Absorbance at an optical density (OD) of 450 nm and 570 nm was measured on a Synergy2 plate reader (BioTek Instrument). The background 570 nm OD was subtracted from the 450 nm reading.

#### Antibody competition ELISA

96-well half area plates (Corning) were coated at room temperature for 8 h with 1ug/mL PolyRab anti-His antibody (ThermoFisher, PA1-983B), followed by overnight blocking with blocking buffer containing 1x PBS, 5% skim milk, and 0.1% Tween-20. The plates were then incubated with 1ug/mL of his-tagged BG505.MD39 at room temperature for 1 h. HIV antibodies were serially diluted 3-fold (starting concentration,100 ug/mL) with blocking buffer and incubated on the plates for 2 h. A no competitor control used dilution buffer at this step. C24 and C33 antibodies were biotinylated using Novus Biologicals Lightning-Link rapid type A Biotin antibody labeling kit (NovusBio, 370-0010) according to protocol. The biotinylated antibodies were added to wells at a concentration of 1 ug/ml (for C24) or 10 ug/mL (for C33) and incubated at RT for 1 h. The plates were further incubated at room temperature for 1 h with native streptavidin-HRP (Abcam, ab7403) at 1:15,000 dilution, followed by the addition of TMB substrate (ThermoFisher) and then quenched with 1 M H2SO4. Absorbance at 450 nm and 570 nm were recorded with a BioTek plate reader. Four washes were performed between every incubation using PBS with 0.05% Tween.

### Surface plasmon resonance binding measurements

Antibody binding measurements were performed using a Biacore 8k instrument (GE). Antibodies were captured on a Series S Sensor Protein A capture chip (Cytiva) with approximately 200–300 RUs of IgG captured on each flow cell at a flow rate of 10 μL/min for 60 s. Running buffer was HBS-EP (3 M sodium chloride/200 mM HEPES/60 mM EDTA/1.0% Tween 20 pH = 7.6) (Teknova) with 0.1% (w/v) bovine serum albumin. All measurements were performed at 25 °C. Each run had 3 startup cycles with 60 s contact time at a flow rate of 50 μL/min. A monomeric BG505 gp120 protein sample was diluted in running buffer and flowed across the chip after capturing at a rate of 50 μL/min. A three-fold dilution series was made starting from 500 nM for higher affinity antibody, C24, or a six-fold dilution series starting from 5000 nM for lower affinity antibody, C33. Experiments were reference-subtracted and run as single-cycle kinetics with a 1:1 binding model using Biacore Evaluation software. The experiment had a 120 s contact time phase and 600 s dissociation phase. Regeneration was performed with 10 mM glycine at pH = 1.5 at a flow rate of 50 µL/min for 30 s after each cycle. The IgG binding data yields an apparent K_D_ due to avidity.

### Neutralization assay

Pseudotyped viruses were titered to yield 150, 000 RLU after 48 h of infection with TZM-BL cells^74^. Mouse serum was heat-inactivated for 15 min at 56 °C. Serum or monoclonal antibody controls were serially diluted in 96-well plates and incubated with a pseudotyped virus before adding 10,000 TZM-BL cells (NIH AIDS Reagent Program) per well with dextran (ThermoFisher). Forty-eight hours after incubation, media was removed and cells were lysed using BriteLite luciferase reagent (Promega). Luminescence was then measured using the Synergy2 plate reader (BioTek Instruments). Serum titer was determined for 50% virus neutralization (ID_50_).

### Mouse IFN-gamma Enzyme-linked immunospot assay (ELISpot)

Ninety-six well filter plates were pre-coated with anti-IFN-γ capture antibody (MabTech). Spleens were isolated from mice 2 weeks after the final immunization. After processing the spleens to obtain a single-cell suspension, 2 × 10^5^ cells were added to the blocked plates. Cells were stimulated with overlapping 15mer peptide pools for WT BG505 gp160 (5 ug/ml per peptide—GenScript). Media alone and concanavalin A (Invitrogen) were used as negative and positive controls respectively. After 18 h of stimulation at 37 °C, the plates were washed, and the detection antibody (R4-6A2-biotin) was added for 2 h at RT. Plates were then washed and the Streptavidin-ALP antibody (1:1000) was added for 1 h at RT. Plates were then developed using the BCIP/NBT-plus for 10 min. Plates were then scanned and counted using CTL-ImmunoSpot^®^ S6 FluoroSpot plate reader (Cellular Technology Limited CTL).

### Flow-cytometry-based assays

#### Intracellular cytokine staining

For intracellular cytokine staining, 2 × 10^6^ splenocytes were stimulated in the presence of protein transport inhibitor, GolgiStop^TM^ GolgiPlug^TM^ (BD Bioscience) with the same peptide pools as the ELISpots. Media alone and phorbol 12-myristate 13-acetate (PMA) and ionomycin stimulations (BD Bioscience) were used as negative and positive controls respectively. To test for degranulation of cells, FITC anti-CD107a (Biolegend) antibody was also added during stimulation. After 6 h, cells were washed and stained with LIVE/DEAD violet. Surface staining was then added containing BV510 anti-CD4, APC-Cy7 anti-CD8, BV711 anti-CD62L, and AF700 anti-CD44 (Biolegend). After 30 min incubation, cells were spun, washed, and fixed using the CytoPerm CytoWash kit following the manufacturer’s protocol (BD Bioscience). Intracellular staining was then prepared using APC anti-IFNγ, BV650 anti-TNFα, PE-Cy7 anti-IL-2, and PE-Cy5 anti-CD3 (Biolegend). All data were collected on a modified LSRII flow cytometer (BD Bioscience) with FACS DIVA 6.0 GUI followed by analysis with FlowJo software 9.0 (BD Bioscience).

#### Tfh and GC B-cell staining

Single-cell suspension was generated from the spleen as described in the prior section, and from the lymph nodes by applying pressure on the tissues to pass through 40 um strainers. Single-cell suspensions were washed once with PBS, and then stained with live dead dye (fluorescent violet reactive, Thermo Fisher) diluted 333-fold in PBS at room temperature for 10 min, followed by another wash in PBS. The cells were then incubated with mouse Fc-block (anti-mouse CD16/32, BioLegend) in 1% FBS/PBS at room temperature for an additional 5 min, followed by incubation with antibody mixtures (anti-mouse GL7-FITC, anti-mouse CD4 BV510, anti-mouse CD44 AF700, anti-mouse PD1 PE-Cy7, and anti-mouse CXCR5 biotin, BioLegend, each diluted 1:200 in 1% FBS/PBS) without washing for an additional 40 min at room temperature. The cells were washed and then incubated with Streptavidin-APC at 0.5ug/mL in 1% FBS/PBS for 20 min at room temperature. The cells were then washed again and resuspended in 1% FBS/PBS for flow analysis with an LSRII 18-color instrument.

#### Antigen-specific B-cell sorting

To generate FITC and PE labelled MD39 tetramers, biotinylated avi-tagged MD39 produced as previously mentioned were incubated with molar ratios of Streptavidin-FITC or Streptavidin-PE (BioLegend) for 30 min at room temperature^8,56^. Single-cell suspensions from spleen or lymph nodes of the animals were first washed with PBS, then stained with live dead dye (fluorescent violet reactive, Thermo Fisher) diluted in PBS at room temperature for 10 min, followed by the addition of mouse Fc-block (anti-mouse CD16/32, BioLegend) diluted 1:100 in 1% FBS/PBS and incubation at room temperature for additional 30 min. The cells were washed, and then incubated with 5ug/mL Streptavidin-MD39-FITC and Streptavidin-MD39-PE at room temperature for 45 min. Antibody mixtures (anti-mouse CD19-PE-Cy7, Anti-mouse IgD APC-Cy7, and Anti-mouse IgM BV711, BioLegend, each diluted 1:200 in 1% FBS/PBS) were then added to the cells without any intermediate washing steps, followed by incubation for another 45 min at room temperature. The cells were washed once with 1% FBS/PBS, and then resuspended at 10 million/mL concentration in 1% FBS/PBS for sorting with the FACS ARIA II instrument. CD19 + IgM− IgD− MD39-Tetramer-FITC + MD39-Tetramer-PE + cells were then sorted in bulk into 1.5 mL Eppendorf tube containing 1% FBS/PBS for downstream cDNA prep with 10× genomics.

### cDNA library prep of sorted B-cells with 10× genomics, enrichment of heavy and light chain sequences, and next-generation sequencing

Sorted and viable single B cells from each mouse sample were uniquely barcoded using the 10× chromium single-cell platform, and complementary DNA (cDNA) libraries were prepared from enriched mouse VDJ regions for Next Generation Sequencing according to the manufacturer’s protocol (Chromium Next GEM Single Cell V(D)J Reagent Kits v1.1, 10× Genomics, USA). Cell suspensions of each sample, reverse transcription master mix, and partitioning oil were loaded on a lane of a single-cell “G” chip with a targeted cell output of 2000 cells per library and then run on the Chromium Controller. Reverse transcription was performed within the droplets at 53 °C for 45 min and newly synthesized cDNA was amplified for 16 cycles on a Veriti Thermal Cycler (Thermofisher, USA). cDNA size selection was performed using SPRIselect beads (Beckman Coulter, USA) at a ratio of SPRIselect reagent volume to sample volume of 0.6. cDNA was analyzed on an Agilent Bioanalyzer High Sensitivity DNA chip (Agilent, USA) for qualitative and quantitative control purposes. Enrichment of the mouse V(D)J region was done using primers targeting the Illumina P5 oligo sequence and the mouse constant region. Two rounds of targeted PCRs were done at 67 °C for 30 min. for 6 and 8 cycles, respectively. SPRIselect bead clean-up was done in between and after the V(D)J enrichment PCRs. V(D)J enriched cDNA was analyzed on an Agilent Bioanalyzer High Sensitivity DNA chip (Agilent, USA) for qualitative and quantitative control purposes. V(D)J enriched cDNA was fragmented using the proprietary fragmentation enzyme blend for 2 min at 32 °C, followed by end-repair and A-tailing at 65 °C for 30 min. Sequencing adaptors were ligated to the cDNA at 20 °C for 15 min. and after a round of post-ligation SPRIselect bead clean-up, V(D)J enriched cDNA was amplified for nine cycles using a sample-specific index oligo as a primer. A final round of size selection using SPRIselect beads followed. Final library size and quantity were determined using an Agilent Bioanalyzer High Sensitivity DNA chip and a Qubit dsDNA High Sensitivity Assay kit (Thermofisher, USA), respectively. Additional library quantification was done using the Kapa Library Quantification kit for Illumina Libraries (Roche, USA). cDNA libraries were sequenced on a NextSeq 500 Illumina platform using the 300 bp Mid Output sequencing kit (Illumina, USA), at a sequencing configuration of 150 base pair (bp) on read1 and 150 bp on read2.

### Modeling Murine Fabs

Structural models of C05, C24, and C33 were generated using an anti-citrullinated protein antibody (PDB-ID: 5mu0) that uses the same germline pair (IGHV6-6/IGKV3-2) as a template. Amino acids sequences of C05, C24, and C33 were each aligned to the 5mu0 Fab using default alignment algorithm in Geneious software. A Rosetta fixed backbone simulations employing the beta score function was performed to mutate positions with different SHM changes in C05, C24, and C33. The variable loops were modeled using Rosetta Remodel to the appropriate sequences for C05, C24, and C33. Finally, Rosetta Relax protocol was performed using backbone constraints to minimize and polish the models.

### Complexing of HIV peplomer with C05 Fab and Cryo-electron microscopy specimen preparation and data collection

Murine variable chains were cloned onto human constant IgG domains and expressed in Expi293F cells following the recombinant antibody purification protocol described above. The purified antibodies were digested by the use of papain (Sigma). The resulting Fab/Fc mix was affinity purified using sepharose-protein A beads (GE Healthcare); the flow-through was collected and concentrated. Concentration was estimated using an NP80 nanospectrophotometer (Implen). A version of BG505.MD39^32,56^ was expressed in freestyle 293F cells and purified as described above. Complexes between peplomer and C05 Fab were obtained by incubation overnight at 4 C having a 3:1 molar ratio of Fab to the epitope (9:1 Fab to peplomer). The resulting complex was purified by SEC on a 24-mL S6i column (GE Healthcare) mounted on an FPLC (BioRad) and concentrated. The complex was vitrified (vitrobot Mark IV, FEI) on an in-house-functionalized UltrAUFoil grid (Quantifoil; 300 mesh, 1.2/1.3). The vitrified specimen was introduced into a Titan Krios electron microscope (FEI) with an energy filter and a K3 direct electron detector (Gatan-Ametek). Data was collected overnight employing an aberration-free beam/image shift protocol (EPU, FEI). Data processing was performed employing a standard cryo-EM data processing workflow comprising motion correction, spectral signal weighting and summation, reference-free LoG picking, 2D classification, manual inspection/selection of 2D class averages, and associated molecular projection image data and asymmetric 3D refinement (Relion v3.1)^75^. The resulting density map had an estimated resolution of 3.8 Å using a data half-set inner-consistency FSC cut-off of 0.143.

### Model building and refinement

A homology model of our BG505.MD39 version was obtained using modeler^76^ and a homology model of C05 Fab was obtained using Rosetta. Homology models were rigid-body docked into our density map (UCSF Chimera)^77^. The resulting system was manually inspected in Coot and rebuilt where necessary^78^. The entire HCDR3 region of C05 was rebuilt guided by our density map. The resulting protein build was refined in Rosetta by in-house developed protocols. N-linked glycans were added manually in Coot and four iterations of refinement (Rosetta) and manual model adjustment (Coot) commenced resulting in the final model being deposited in the PDB. Model geometry was validated with MolProbity oneline-analysis^79^, glycan geometry passed validation using Privateer^80^ and model-to-map fit was evaluated with EMringer^81^.

### Statistical analysis

All statistics and calculations were performed using GraphPad Prism 8.0. EC_50_ and EC_70_ concentrations were calculated using a nonlinear regression model. IC_50_ values were computed with a nonlinear regression model of percentage neutralization vs log reciprocal serum dilution. Power analysis was performed with R based on our preliminary data to determine the smallest sample size that would allow us to achieve a power of 0.9 with a pre-set α-value of 0.05. Each individual data point was sampled independently. Two-tailed Mann–Whitney Rank tests were used to compare differences between groups with correction for multiple comparisons. *P*-values were all calculated in GraphPad PRISM, and *p*-values less than 0.05 are considered significant.

### Reporting summary

Further information on research design is available in the Nature Research Reporting Summary linked to this article.

## Supplementary information

Supplementary information

Reporting Summary

## Data Availability

The Cryo-EM data generated in this study have been deposited in the PDB database under accession code 7SQ1. All raw data generated in this paper are available upon request to the corresponding author D.W.K. (dwkulp@wistar.org). Source data are provided with this paper.

## Source data

Source Data

## Materials availability

All reagents generated in this paper are available upon request to the corresponding author D.W.K. (dwkulp@wistar.org).
