# Supplementary material for: Induction of tier-2 neutralizing antibodies in mice with a DNA-encoded HIV envelope native like trimer
Source: Nat Commun. 2022 Feb 4;13:695. doi: 10.1038/s41467-022-28363-z (PMC8816947; doi:10.1038/s41467-022-28363-z)
Supplement: Supplementary file 1 — Supplementary information [file 41467_2022_28363_MOESM1_ESM.pdf]

Induction of tier-2 neutralizing antibodies in mice with a DNA-encoded HIV envelope native like trimer

Ziyang Xu[1,2#], Susanne Walker[1#], Megan C. Wise[3#], Neethu Chokkalingam[1#], Mansi Purwar[1#], Alan Moore[4#], Edgar Tello-Ruiz[1], Yuanhan Wu[1], Sonali Majumdar[1], Kylie M. Konrath[1,2], Abhijeet Kulkarni[1], Nicholas J Tursi[1,2], Faraz I. Zaidi[1], Emma L. Reuschel[1], Ishaan Patel[1], April Obeirne[1], Jianqiu Du[4], Katherine Schultheis[3], Lauren Gites[3], Trevor Smith[3], Janess Mendoza[3], Kate E. Broderick[3], Laurent Humeau[3], Jesper Pallesen[4], David B. Weiner[1], Daniel W. Kulp[1*]

[1] Vaccine and Immunotherapy Center, The Wistar Institute, Philadelphia, PA 19104

[2] Perelman School of Medicine, University of Pennsylvania, Philadelphia, PA 19104.

[3] Inovio Pharmaceuticals, Plymouth Meeting, PA, 19462

[4]Molecular and Cellular Biochemistry, Indiana University, Bloomington, IN, 47405

*Corresponding Author: dwkulp@wistar.org

[#]These authors contributed equally to this work

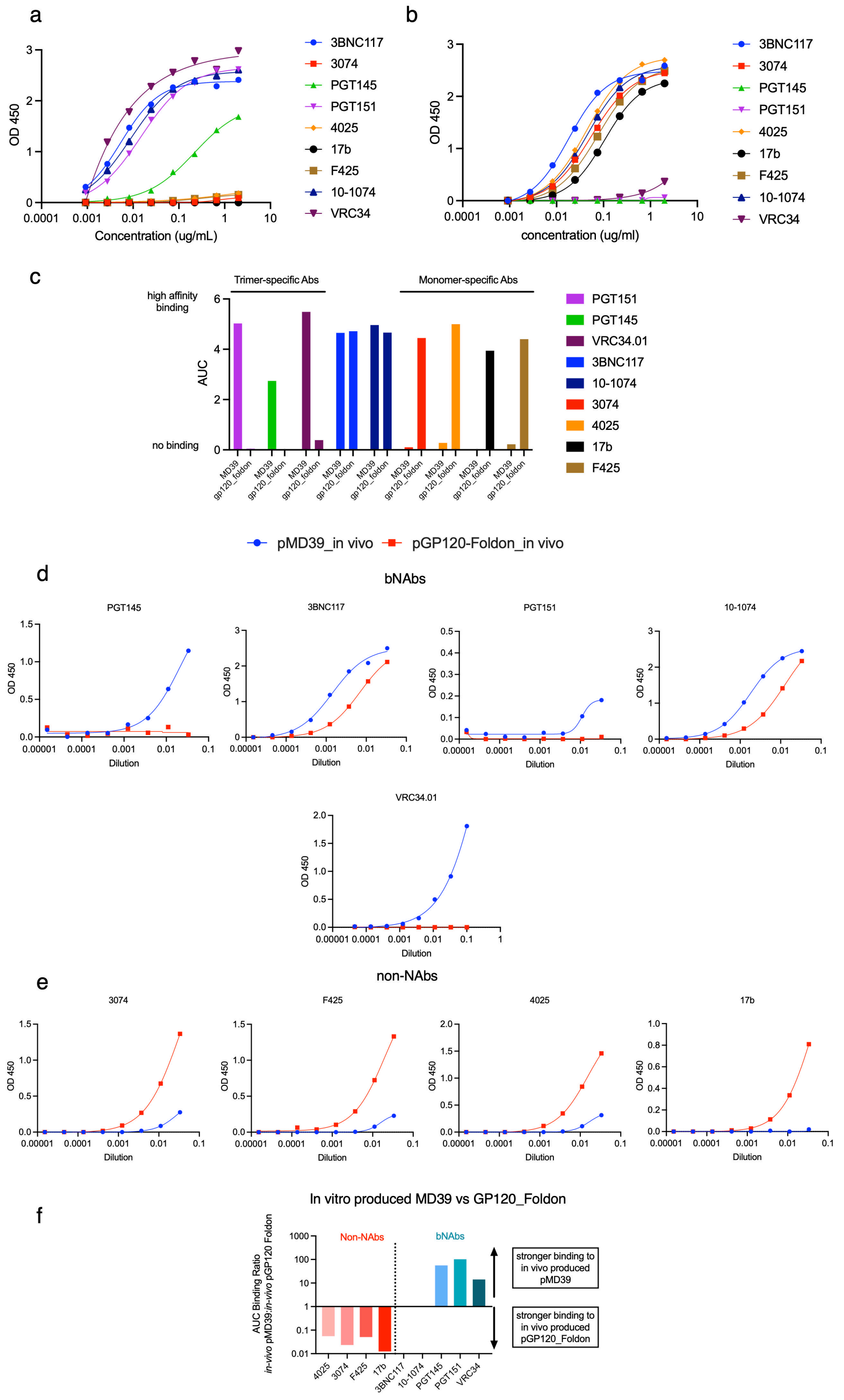
a
OD 450
Concentration (ug/mL)
3BNC117
3074
PGT145
PGT151
4025
17b
F425
10-1074
VRC34
b
OD 450
concentration (ug/ml)
3BNC117
3074
PGT145
PGT151
4025
17b
F425
10-1074
VRC34
c
Trimer-specific Abs
Monomer-specific Abs
high affinity binding
no binding
AUC
MD39
gp120_foldon
PGT151
PGT145
VRC34.01
3BNC117
10-1074
3074
4025
17b
F425
pMD39_in vivo
pGP120-Foldon_in vivo
d
bNAbs
PGT145
3BNC117
PGT151
10-1074
VRC34.01
OD 450
Dilution
e
non-NAbs
3074
F425
4025
17b
OD 450
Dilution
f
In vitro produced MD39 vs GP120_Foldon
AUC Binding Ratio
in-vivo pMD39:in-vivo pGP120 Foldon
Non-NAbs
bNAbs
stronger binding to in vivo produced pMD39
stronger binding to in vivo produced pGP120_Foldon
4025
3074
F425
17b
3BNC117
10-1074
PGT145
PGT151
VRC34

Supplementary Figure 1. Full antigenic profile of *in vitro* expressed MD39 vs BG505.gp120_foldon (related to Figure 1). (**A**) Binding of MD39 to HIV bNAbs PGT145, PGT151, VRC34.01, 3BNC117, 10-1074 or non-NAbs F425, 3074, 4025, 17B. (**B**) Binding of BG505.gp120_foldon to HIV bNAbs and non-NAbs as in (**A**). (**C**) Quantification of binding of MD39 or BG505.gp120_foldon to HIV bNAbs or non-NAbs in terms of Area Under Curve (AUC). (**D** and **E**) Binding of *in vivo* produced MD39 (blue) and GP120-foldon (red) to bNAbs PGT145, 3BNC117, PGT151, 10-1074 and VRC34.01 (**D**) and non-NAbs 3074, F425, 4025 and 17b (**E**). (**F**) Normalized area under the curve (ratio of *in vitro* produced pMD39 binding versus pGP120-foldon binding) for binding to HIV bNAbs and non-NAbs.

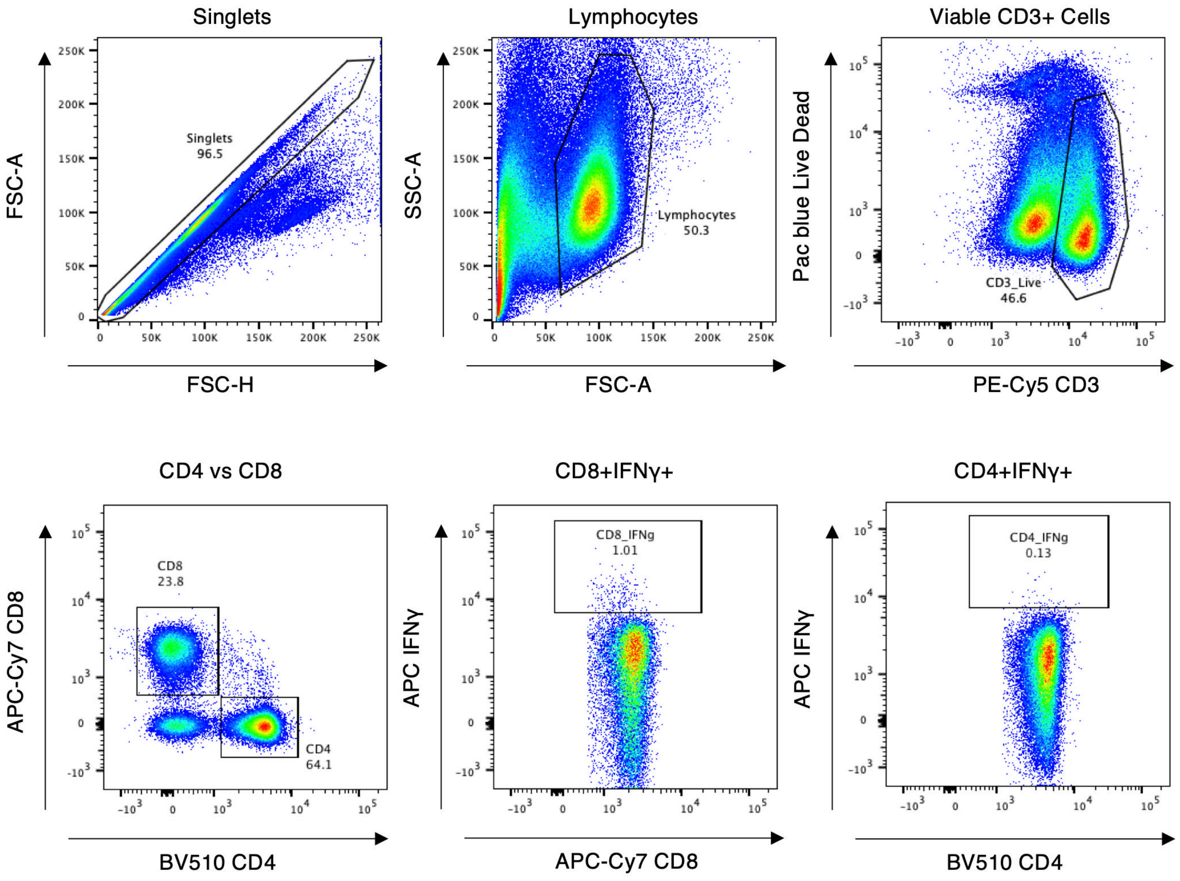

Supplementary Figure 2. ICS Gating scheme. Gating scheme used to determine cytokine positive $CD4^{+}$ or $CD8^{+}$ T cells following peptide stimulation **(related to Figure 2 and 3).**

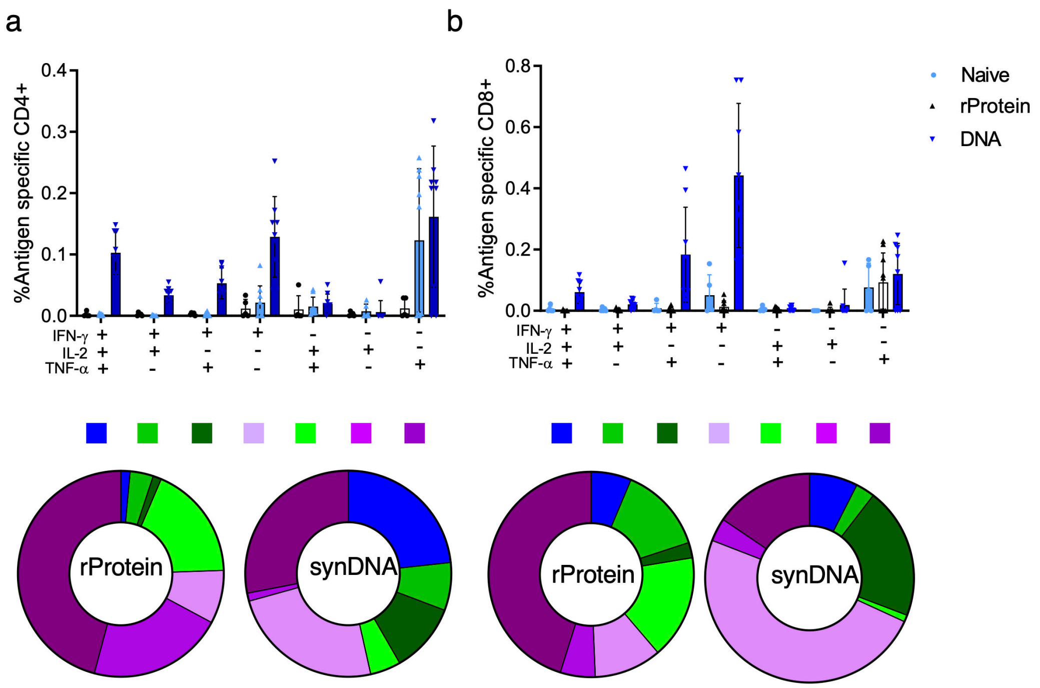

Supplementary Figure 3. DNA-encoded BG505.MD39 but not protein BG505.MD39 induced polyfunctional T-cell responses in BALB/c mice (related to Figure 2). Mice were immunized with 25ug of either DNA-encoded BG505.MD39 or RIBI-co-formulated protein BG505.MD39 at weeks 0, 3, 6 and euthanized at week 8 for cellular analysis. (**A** – **B**) ICS analyses of co-expression of IFNγ, TNFα and IL-2 cytokines in CD4^{+} (**A**) or CD8^{+} (**B**) T cells following stimulation with BG505 Envelope specific peptide pools. Two independent experiments were performed for each panel in the figure with similar findings. N=10 mice/group. Error bar represents standard deviation. Center of the error bar represents the mean. Source data are provided as a Source Data file.

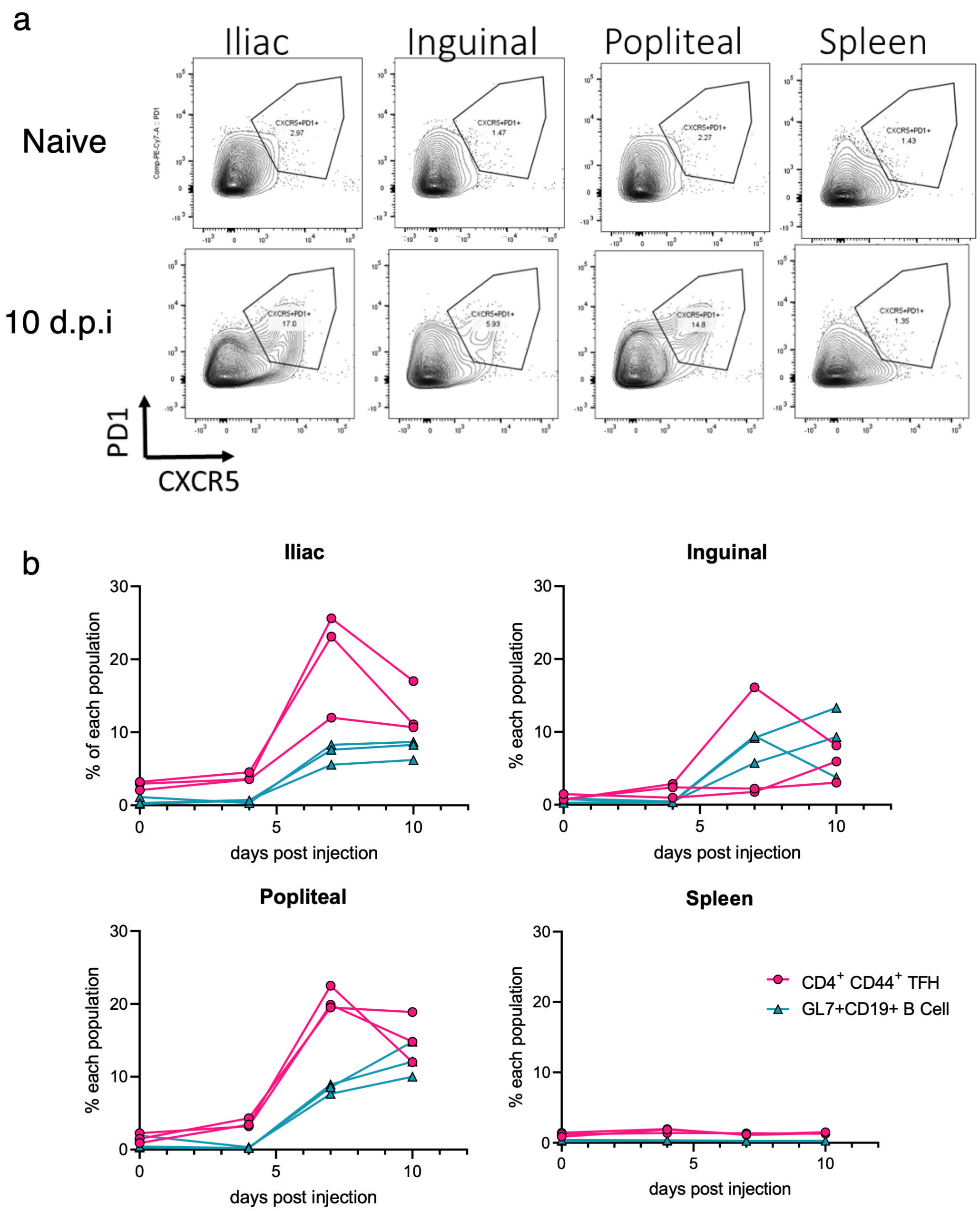

Supplementary Figure 4. DNA-encoded BG505.MD39 vaccination induced both Tfh and GC B cell responses in BALB/c mice (related to Figure 2). Mice were immunized with 25ug of DNA-encoded BG505.MD39 on Day 0 and euthanized sequentially on Days 4, 7 and 10 for analyses of Tfh and GC B cells. (**A**) Comparison of flow plots of CXCR5+PD1+ cells amongst CD4+CD44+ cells in the iliac, inguinal, popliteal and spleen of naïve mice versus mice immunized with DNA-encoded BG505.MD39 10 d.p.i. (**B**) Kinetics of Tfh and GC B cell responses in the draining lymph nodes or spleens of mice vaccinated with DNA-encoded

BG505.MD39. One independent experiment was performed for each panel in the figure. Each line represents an animal. N=3 mice/group. Source data are provided as a Source Data file.

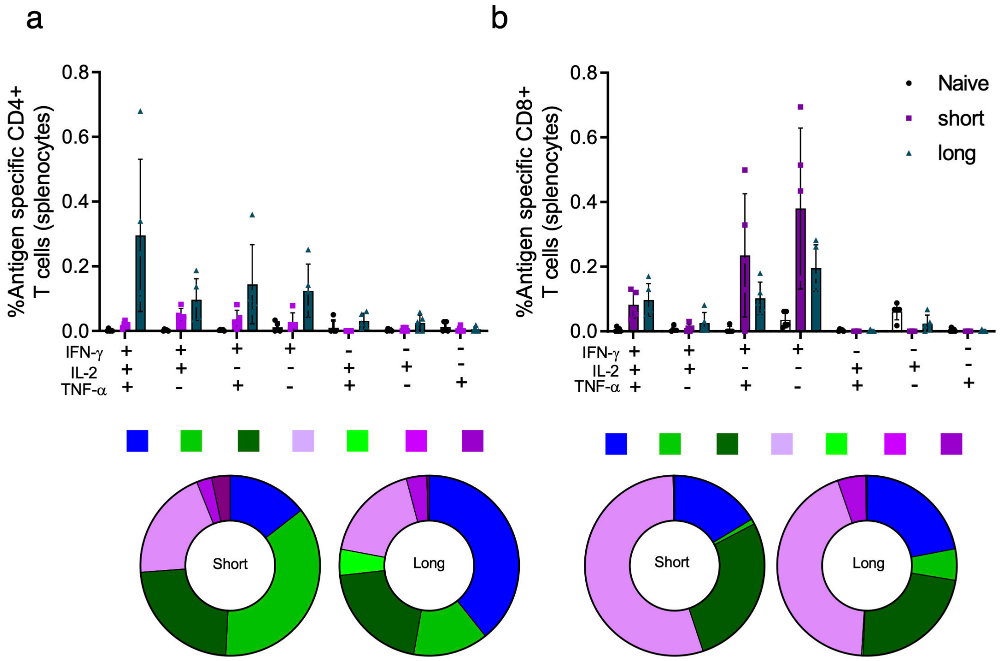

Supplementary Figure 5. Long immunization scheme with DNA-encoded BG505.MD39 improved polyfunctional CD4⁺ T-cell responses, while short immunization scheme improved polyfunctional CD8⁺ T-cell responses in mice (related to Figure 3). Mice in this panel were immunized either with a short scheme (Wks 0, 3, 6) or a long scheme (Wks 0, 3, and 16) with 25ug DNA used at each vaccination. The mice were euthanized 2 weeks post the final vaccination for cellular analyses. (**A** – **B**) ICS analyses of co-expression of IFNγ, TNFα and IL-2 cytokines in $CD4^+$ (**A**) or $CD8^+$ (**B**) T cells following stimulation with BG505 Envelope specific peptide pools in mice immunized with DNA-encoded BG505.MD39 using either the short or the long scheme. Two independent experiments were performed for each panel in the figure with similar findings. N=5 mice/group. Error bar represents standard deviation. Center of the error bar represents the mean. Source data are provided as a Source Data file.

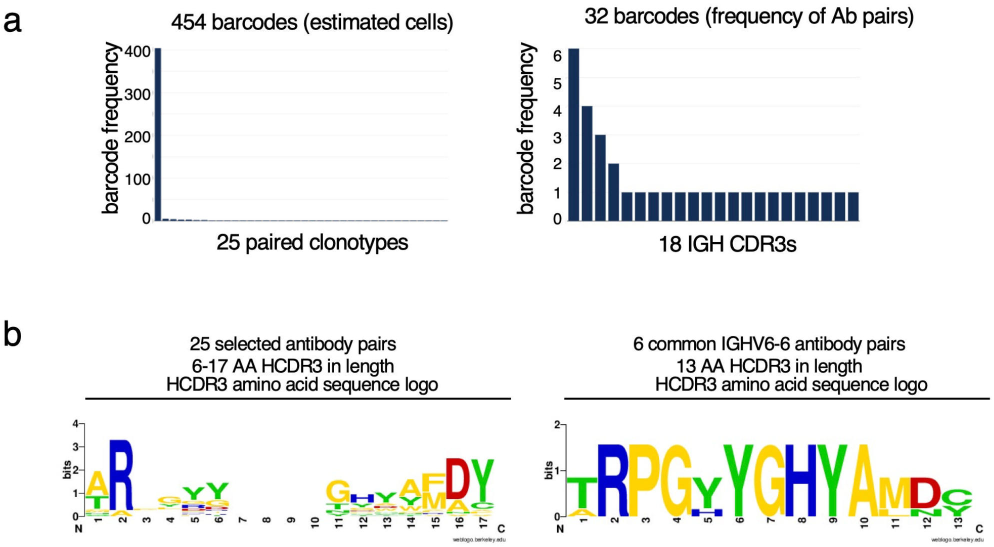

Supplementary Figure 6. Sequence features of recovered murine antibody clones (related to Figure 5). (**A)** Demonstration of the barcode frequencies by clones (Left) or IGH HCDR3 identities (Right). (**B)** HCDR3 features of the 25 recovered MAb clones (left) or the 6 clones with IGHV6-6 germline VH gene usage (Right).

a

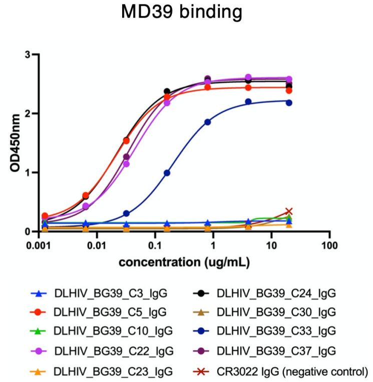

b

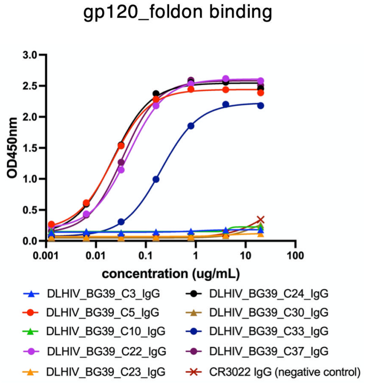

c

EC_{50} (ng/mL)	MD39	gp120
C3	>20,000	>20,000
C5	21.94	20.53
C10	>20,000	>20,000
C22	44.97	36.54
C23	>20,000	>20,000
C24	22.05	21.87
C30	>20,000	>20,000
C33	203.7	72.28
C37	34.71	29.27
4025	>20,000	4.685
3074	>20,000	4.29
CR3022	>20,000	>20,000

d

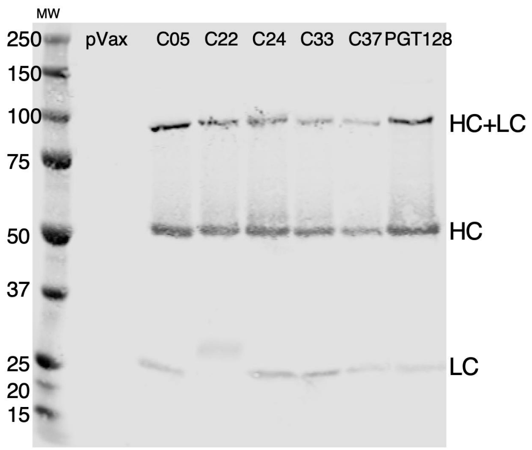

e

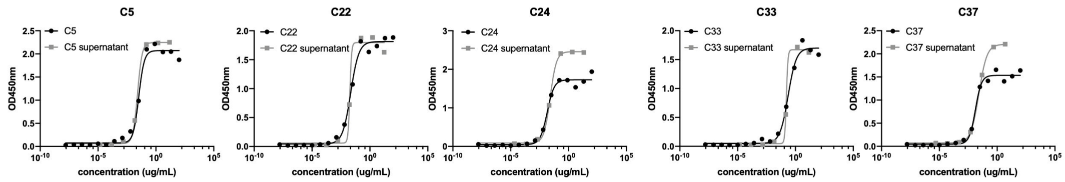

Dilution Factor	Calculated Concentration (ug/ml)				
	C5	C22	C24	C33	C37
1	15	17	21	14.5	5
100				0.145	
1000	0.015	0.017	0.021		0.005

f

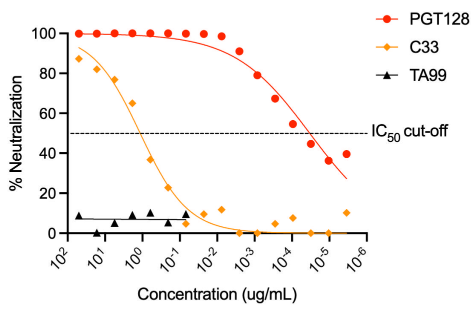

g

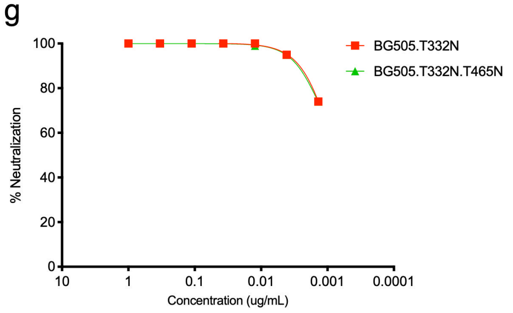

Supplementary Figure 7. Further characterizations of BG505.MD39-specific murine antibody clones (related to Figure 6). (**A-C**) Binding of all isolated murine mAb clones to BG505.MD39 (**A**) or gp120_foldon (**B**) as determined by ELISA, and respective measured EC_{50} values (**C**). (**D**) Reducing SDS PAGE analysis of BG505.MD39-specific MAb clones C05, C22, C24, C33, and C37 in comparison with PGT128 or backbone pVAX vector transfection supernatants. (**E**) Determination of the antibody concentrations from the transfection supernatants based on BG505.MD39 binding in comparison with purified protein standards. (**F**) Neutralization of autologous BG505.T332N virus by PGT128 (positive control), C33, and murine IgG2A antibody TA99 (negative control) starting at 50ug/mL. (**G**) Comparison of neutralization potential of PGT128 with BG505.T332N pseudo-virus versus BG505.T332N.T465N pseudo-virus. For (D), the experiments were repeated twice.

a

AA variances	C5 HC	C22 HC	C24 HC	C33 HC	C37 HC
C5 HC	0	15	14	10	12
C22 HC	15	0	14	11	13
C24 HC	14	14	0	8	11
C33 HC	10	11	8	0	7
C37 HC	12	13	11	7	0

AA Variances: 0, 5, 10, 15

AA variances	C5 Kap	C22 Kap	C24 Kap	C33 Kap	C37 Kap
C5 Kap	0	11	7	5	5
C22 Kap	11	0	4	6	9
C24 Kap	7	4	0	2	5
C33 Kap	5	6	2	0	5
C37 Kap	5	9	5	5	0

AA Variances: 0, 5, 10, 15

b

Heavy chain mutations away from germline

			CDR1			CDR2														CDR3				
Amino Acid Position	6	18	24	32	33	35	58	59	61	64	68	71	75	82	84	86	88	90	92	94	103	107	113	114	115
HV6-6+HD1-2+HJ4	V	P	L	T	F	N	K	S	N	T	E	K	T	K	S	Y	Q	N	L	A	T	Y	M	D	Y
c5	V	A	L	S	L	N	K	P	N	T	E	K	S	R	T	Y	Q	N	L	A	T	Y	M	D	Y
c22	V	P	I	S	F	S	K	P	S	T	E	K	T	K	S	Y	E	S	V	V	A	H	M	D	C
c24	E	P	L	T	F	S	P	P	N	T	D	R	T	K	S	Y	Q	N	L	T	T	Y	M	N	C
c33	V	P	L	T	F	S	K	S	N	T	E	K	T	K	S	Y	Q	N	L	A	T	Y	L	D	C
c37	V	P	I	T	F	S	R	A	N	S	E	K	T	K	S	D	Q	N	L	A	T	Y	M	D	Y

Light chain mutations away from germline

			CDR1		CDR2							
Amino Acid Position	15	25	34	35	54	58	62	67	85	87	89	108
KV3-2+KJ2	L	A	I	S	A	Q	V	S	D	T	M	L
c5	P	P	I	S	A	R	V	T	D	I	M	L
c22	L	A	V	S	G	Q	A	S	N	T	V	V
c24	L	A	V	S	G	Q	V	S	D	T	M	L
c33	L	A	I	S	A	Q	V	S	D	T	M	L
c37	P	A	V	N	A	Q	V	T	D	P	M	L

Supplementary Figure 8. Additional sequence features of recovered murine antibody clones (related to Figure 6). Comparisons of the heavy and light chain sequences of the 5 BG505.MD39-specific MAb clones by number of mutations (**A**) and positions for the mutations (**B**).

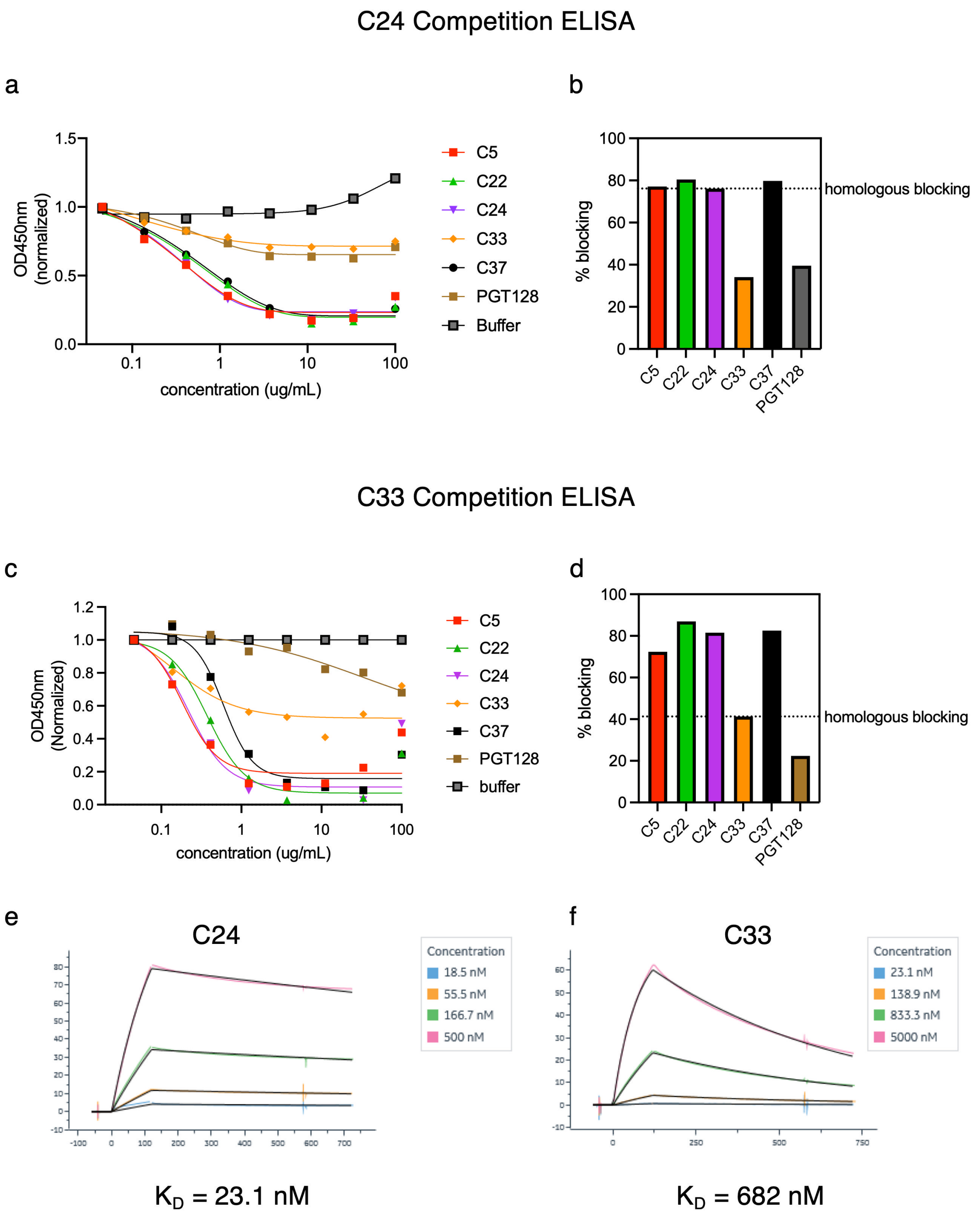

Supplementary Figure 9. Assessment of epitope specificity and affinities of C24 and C33 by competition ELISA and SPR (related to Figure 6). (**A**) Degree of binding of biotinylated C24 to MD39 in the presence of varying concentrations of non-biotinylated C05, C22, C24, C33, C37 and control buffer. (**B**) Degree of blocking of biotinylated C24 binding to MD39 by each of the aforementioned antibodies, as calculated

from (**A**). (**C**) Degree of binding of biotinylated C33 to MD39 in the presence of varying concentrations of non-biotinylated C05, C22, C24, C33, C37 and control buffer. (**D**) Degree of blocking of biotinylated C33 binding to MD39 by each of the aforementioned antibodies, as calculated from (**C**). (**E** and **F**) SPR trace to determine the binding and dissociation pattern of C24 (**E**) and C33 (**F**) to GP120 (to preserve 1:1 binding ratio). For (**A**-**D**), two technical replicates were determined for each experimental condition; error bar represents standard deviation. Source data are provided as a Source Data file.

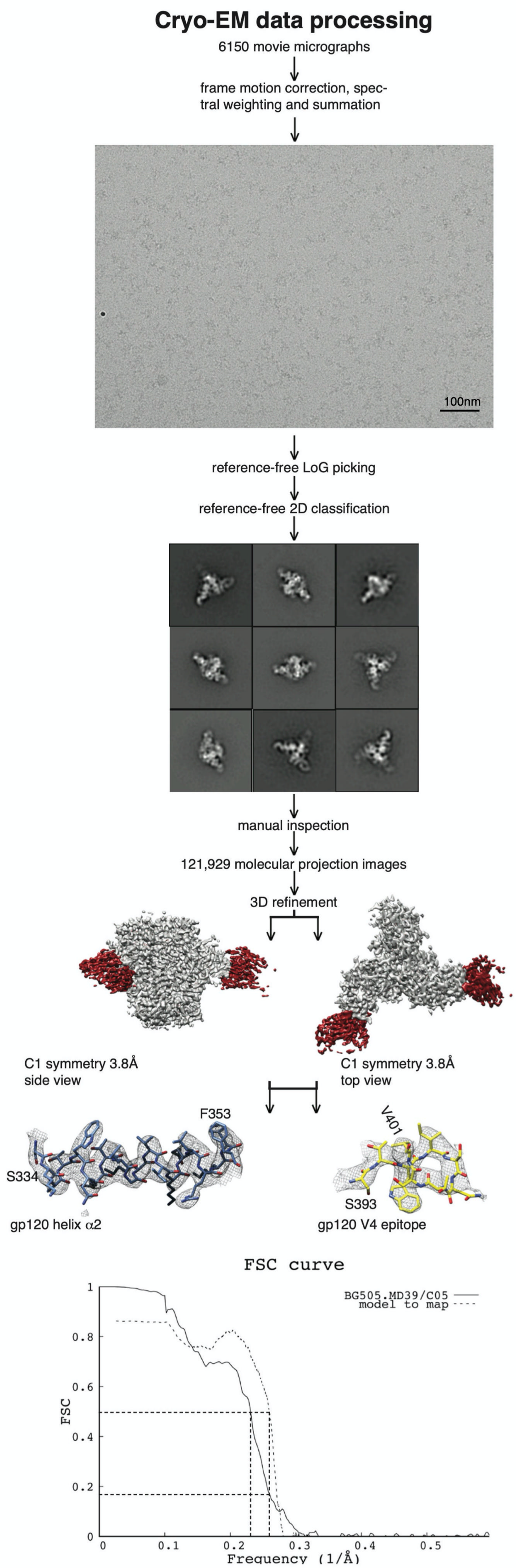
Cryo-EM data processing
6150 movie micrographs
frame motion correction, spectral weighting and summation
100nm
reference-free LoG picking
reference-free 2D classification
manual inspection
121,929 molecular projection images
3D refinement
C1 symmetry 3.8Å side view
C1 symmetry 3.8Å top view
F353
S334
gp120 helix α2
V401
S393
gp120 V4 epitope
FSC curve
BG505.MD39/C05
model to map
FSC
Frequency (1/Å)
1
0.8
0.6
0.4
0.2
0
0
0.1
0.2
0.3
0.4
0.5

Supplementary Figure 10. Cryo-EM data processing flow diagram resulting in a density map at 3.8Å global resolution (FSC 0.143 consistency between two independently refined data half sets) (related to Figure 7). Flow diagram comprises panels of representative map densities with corresponding atomic models superimposed as well as a standard Fourier shell correlation plot (FSC plot). MD39 is shown in grey; C05 Fab is shown in dark red.

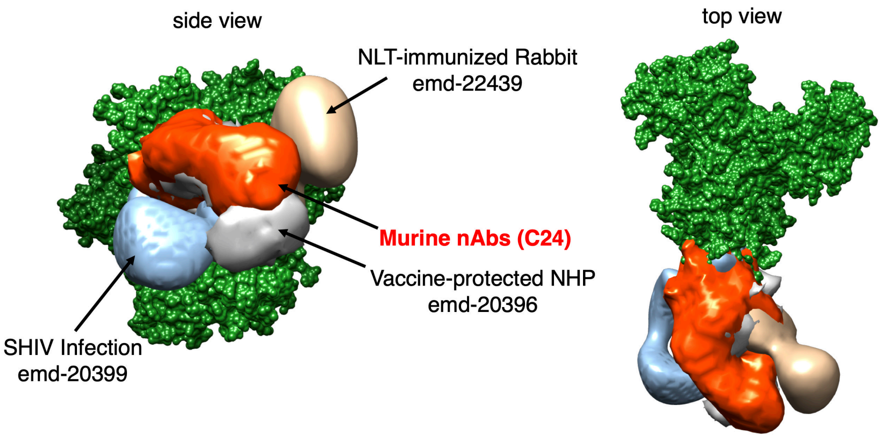

Supplementary Figure 11. Computational modeling of murine mAbs bound to NLT. Comparison of the angle of approach in terms of binding to NLT between murine and NHP V5-directed mAbs **(related to Figure 7).**

Supplementary Table 1. Comparison of neutralizing antibody titers in mice immunized with DNA-encoded NLT versus protein MD39. BG505.T332N or MLV ID50 neutralization titers in the sera of animals immunized with 25ug RIBI co-formulated protein BG505.MD39 or DNA-encoded BG505.MD39 at Weeks 0, 3, 6 two weeks post the final vaccination.

	Mouse ID	BG505.T332N	MLV
Protein	1	<45	<45
	2	<45	<45
	3	<45	<45
	4	<45	<45
	5	<45	<45
	6	<45	<45
	7	<45	<45
	8	<45	<45
	9	<45	<45
	10	<45	<45
DNA	1	<45	<45
	2	<45	<45
	3	232	<45
	4	<45	<45
	5	<45	<45
	6	73	<45
	7	50	<45
	8	<45	<45
	9	<45	<45
	10	<45	<45

Supplementary Table 2. Comparison of neutralizing antibody titers in mice immunized with DNA-encoded NLT using a short versus a long boost scheme. BG505.T332N or MLV ID50 neutralization titers in the sera of naïve mice or animals immunized with 25ug DNA-encoded BG505.MD39 at Weeks 0, 3, 6 (short scheme) or Weeks 0, 3, 16 (long scheme) two weeks post the final vaccination.

	Mouse ID	BG505.T332N	MLV
Naïve	1	<45	<45
	2	<45	<45
	3	<45	<45
	4	<45	<45
	5	<45	<45
Short	1	<45	<45
	2	<45	<45
	3	<45	<45
	4	>1215	<45
	5	83	<45
	6	132	<45
	7	<45	<45
	8	<45	<45
	9	<45	<45
	10	<45	<45
Long	1	>1215	<45
	2	48	<45
	3	>1215	<45
	4	824	<45
	5	<45	<45
	6	194	<45
	7	53	<45
	8	<45	<45
	9	46	<45
	10	<45	<45

Supplementary Table 3. Key statistics of cryo-EM data processing, model building and validation.

Map	BG505.MD39TS/C05
PDB code	7SQ1
EMDB code	25376
Data collection	
Microscope	FEI Titan Krios
Voltage (kV)	300
Detector	Gatan K3
Recording mode	Counting
Magnification (incl. post-magnification; EFTEM)	105,000
Movie micrograph pixelsize (Å)	0.84
Dose rate (e^-/[(camera pixel)*s])	16.372
Number of frames per movie micrograph	60
Frame exposure time (ms)	50
Movie micrograph exposure time (s)	2.59
Total dose (e^-/Å^2)	60.6
Defocus range (μm)	0.4-3.3
EM data processing	
Number of movie micrographs	5,485
Number of molecular projection images in map	121,929
Symmetry	C1
Map resolution (FSC 0.143; Å)	3.8
Map sharpening B-factor (Å^2)	-75
Structure Building and Validation	
Number of atoms in deposited model	18,364
gp120	3,962
gp41	1,025
glycans	1,373
MolProbity score	1.147 (99%)
Clashscore	3.57
EMRinger score	2.37
model to map correlation (FSC 0.5; Å)	3.8
Deviations from ideal	
Bond length outliers (RMSD; Å, #)	0.011 (0)
Bond angles outliers (RMSD; °, #)	1.499 (28)
Ramachandran plot	
Favored (%)	98.77
Allowed (%)	1.18
Outliers (%)	0.04

Supplementary Note 1

Nucleic acid sequence: C05_HC_VH
GAAGTGAAGCTTGAGGAGTCTGGAGGAGGCTTGGTGCAAGCTGGAGGATCCATGAAACTCTCCTGTGTTGCCTCT
GGATTCAGTCTCAGTAATTATTGGATGAACTGGGTCCGCCAGTCTCCAGAGAAGGGGCTTGAGTGGGTTGCTGAA
ATTAGATTGAAGCCTCAAAATTATGCAACACATTATGCGGAGTCTGTGAAAGGGAGGTTCAGCATCTCAAGAGAT
GATTCCAGAAGTACTGTCTACCTGCAAATGAACAACTTAAGAGCTGAAGACACTGGCATTTATTACTGTACCAGGC
CGGGATACTACGGCCACTATGCTATGGACTACTGGGGTCAAGGAACCTCAGTCACCGTCTCCTCA

Nucleic acid sequence: C05_Kappa
GACATTGTGCTGACCCAGTCTCCAGCTTCTTTGGCTGTGTCTCCAGGTCAGAGGGCCACCATCTCCTGCAGACCCA
GCGAAAGTGTTGATAATTACGGCATTAGCTTTATGAACTGGTTCCAACAGAAACCAGGACAGCCACCCAAACTCCT
CATCTATGCTGCATCCAACCGAGGATCCGGGGTCCCTGCCAGGTTTACTGGCAGTGGGTCTGGGACAGACTTCAG
CCTCAACATCCATCCTATGGAGGAGGATGATATTGCAATGTATTTCTGTCAGCAAAGTAAGGAGGTTCCGTATACG
TTCGGAGGGGGGACCAAACTGGAAATAAAA
