# Supplementary material for: Induction of tier-2 neutralizing antibodies in mice with a DNA-encoded HIV envelope native like trimer
Source: Nat Commun. 2022 Feb 4;13:695. doi: 10.1038/s41467-022-28363-z (PMC8816947; doi:10.1038/s41467-022-28363-z)
Supplement: Supplementary file 2 — Reporting Summary [file 41467_2022_28363_MOESM2_ESM.pdf]

nature research

Corresponding author(s): Daniel W. Kulp

Last updated by author(s): Dec 10, 2021

Reporting Summary

Nature Research wishes to improve the reproducibility of the work that we publish. This form provides structure for consistency and transparency in reporting. For further information on Nature Research policies, see Authors & Referees and the Editorial Policy Checklist.

Statistics

For all statistical analyses, confirm that the following items are present in the figure legend, table legend, main text, or Methods section.

n/a	Confirmed	
☐	☒	The exact sample size (*n*) for each experimental group/condition, given as a discrete number and unit of measurement
☐	☒	A statement on whether measurements were taken from distinct samples or whether the same sample was measured repeatedly
☐	☒	The statistical test(s) used AND whether they are one- or two-sided<br>*Only common tests should be described solely by name; describe more complex techniques in the Methods section.*
☒	☐	A description of all covariates tested
☐	☒	A description of any assumptions or corrections, such as tests of normality and adjustment for multiple comparisons
☐	☒	A full description of the statistical parameters including central tendency (e.g. means) or other basic estimates (e.g. regression coefficient) AND variation (e.g. standard deviation) or associated estimates of uncertainty (e.g. confidence intervals)
☒	☐	For null hypothesis testing, the test statistic (e.g. *F*, *t*, *r*) with confidence intervals, effect sizes, degrees of freedom and *P* value noted<br>*Give P values as exact values whenever suitable.*
☒	☐	For Bayesian analysis, information on the choice of priors and Markov chain Monte Carlo settings
☒	☐	For hierarchical and complex designs, identification of the appropriate level for tests and full reporting of outcomes
☒	☐	Estimates of effect sizes (e.g. Cohen's *d*, Pearson's *r*), indicating how they were calculated

Our web collection on statistics for biologists contains articles on many of the points above.

Software and code

Policy information about availability of computer code

Data collection	FACS DIVA 6.0, ROSETTA
Data analysis	FlowJo 9.0, Prism 8.0, Relion v3.1, Coot, UCSF Chimera 1.15

For manuscripts utilizing custom algorithms or software that are central to the research but not yet described in published literature, software must be made available to editors/reviewers. We strongly encourage code deposition in a community repository (e.g. GitHub). See the Nature Research guidelines for submitting code & software for further information.

Data

Policy information about availability of data

All manuscripts must include a data availability statement. This statement should provide the following information, where applicable:

- Accession codes, unique identifiers, or web links for publicly available datasets
- A list of figures that have associated raw data
- A description of any restrictions on data availability

The CryoEM data generated in this study have been deposited in the PDB database under accession code 7SQ1 [https://www.rcsb.org/structure/7SQ1]. Source data are provided with this paper. All raw data generated in this paper are available upon request to the corresponding author Dr. Daniel W. Kulp (dwkulp@wistar.org).

Field-specific reporting

Please select the one below that is the best fit for your research. If you are not sure, read the appropriate sections before making your selection.

☒ Life sciences ☐ Behavioural & social sciences ☐ Ecological, evolutionary & environmental sciences

Life sciences study design

All studies must disclose on these points even when the disclosure is negative.

Sample size	Sample size was selected based upon mean and standard deviations observed in our previous vaccine studies in these animal models (Xu et al., Advanced Science, 2020), and power analysis was subsequently performed with Prism 8.0 to determine the animal numbers required to have a power of 0.8.
Data exclusions	No data was excluded
Replication	All experiments were repeated at least twice independently, with similar findings as demonstrated in the figures.
Randomization	Age and sex matched animals were randomly allocated into each cage by Animal Facility Staff at the start of the experiment; experimental arms were assigned to different cages after randomization.<br>For experiments that did not involve animals, no randomization was performed as all samples were simultaneously tested.
Blinding	Investigators were not blinded; this is because a specific procedure will need to be performed (electroporation after DNA injection, whereas protein immunization will only require subcutaneous injection) which will require scientists to be aware of the group assignment.

Reporting for specific materials, systems and methods

We require information from authors about some types of materials, experimental systems and methods used in many studies. Here, indicate whether each material, system or method listed is relevant to your study. If you are not sure if a list item applies to your research, read the appropriate section before selecting a response.

Materials & experimental systems

n/a	Involved in the study	
☐	☒	Antibodies
☐	☒	Eukaryotic cell lines
☒	☐	Palaeontology
☐	☒	Animals and other organisms
☒	☐	Human research participants
☒	☐	Clinical data

Methods

n/a	Involved in the study	
☒	☐	ChIP-seq
☐	☒	Flow cytometry
☒	☐	MRI-based neuroimaging

Antibodies

Antibodies used

anti-mouse IgG H+L HRP (Bethyl, Catalog: A90-116P), (dil 1:20,000)
anti-mouse CD4-BV510, Biolegend, Catalog: 100559, (dil 1:200)
anti-mouse CD8–APC-Cy7, Biolegend, Catalog: 100714 (dil 1:200)
anti-mouse IL2–PE-Cy7, BioLegend, Catalog: 503832 (dil 1:100)
anti-mouse IFNγ-APC, BioLegend, Catalog: 505810 (dil 1:100)
anti-mouse CD3e–PE-Cy5, BioLegend, Catalog: 100310 (dil 1:100)
anti-mouse TNFα-BV605, BioLegend, Catalog: 506329 (dil 1:100)
anti-mouse CD19-PE-Cy7, BioLegend, Catalog: 115520 (dil 1:200)
Anti-mouse IgD APC-Cy7,BioLegend, Catalog: 405716 (dil 1:200)
Anti-mouse IgM BV711, BioLegend, Catalog: 406539 (dil 1:200)
2G12 antibody, NIH AIDS Reagent, Catalog: 1476, (dil 1:1000)
3074 antibody, NIH AIDS Reagent, Catalog: 12040 (dil 1:1000)
PGT145 antibody, NIH AIDS Reagent, Catalog: 12703 (dil 1:1000)
PGT128 antibody, NIH AIDS Reagent, Catalog: ARP-13352 (dil 1:1000)
F425 antibody, NIH AIDS Reagent, Catalog: ARP-7626 (dil 1:1000)
17b antibody, NIH AIDS Reagent, Catalog: ARP-4091 (dil 1:1000)
10-1074 antibody, NIH AIDS Reagent, Catalog: ARP-12477 (dil 1:1000)
VRC34.01 antibody, CreativeBioLabs, Catalog: N123-VRC34.01 (dil 1:1000)
Goat anti-Human IgG (H+L) Cross-Adsorbed Secondary Antibody, Alexa Fluor 488, Invitrogen, Catalog: A-11013 (dil 1:200)

Validation

anti-mouse CD4-BV510, Biolegend, Catalog: 100559, validation: https://www.biolegend.com/en-us/products/brilliant-violet-510-anti-mouse-cd4-antibody-7991, Statement: FC-Quality Tested
anti-mouse CD8–APC-Cy7, Biolegend, Catalog: 100714, validation: https://www.biolegend.com/en-us/products/apc-cyanine7-anti-mouse-cd8a-antibody-2269?GroupID=BLG279, Statement: FC-Quality Tested
anti-mouse IL2–PE-Cy7, BioLegend, Catalog: 503832, validation: https://www.biolegend.com/en-us/products/pe-cyanine7-anti-mouse-il-2-antibody-8324?GroupID=GROUP24, Statement: FC-Quality Tested
anti-mouse IFNγ-APC, BioLegend, Catalog: 505810, validation: https://www.biolegend.com/en-us/products/apc-anti-mouse-ifn-gamma-antibody-993?GroupID=GROUP24, Statement: FC-Quality Tested

anti-mouse CD3e–PE-Cy5, BioLegend, Catalog: 100310, validation: https://www.biolegend.com/en-us/products/pe-cyanine5-anti-mouse-cd3epsilon-antibody-26, Statement: FC-Quality Tested
anti-mouse TNFα-BV605, BioLegend, Catalog: 506329, validation: https://www.biolegend.com/en-us/products/brilliant-violet-605-anti-mouse-tnf-alpha-antibody-7682?GroupID=GROUP24, Statement: FC-Quality Tested
anti-mouse CD19-PE-Cy7, BioLegend, Catalog: 115520, validation: https://www.biolegend.com/en-us/products/pe-cyanine7-anti-mouse-cd19-antibody-1907?GroupID=BLG10556, Statement: FC-Quality Tested
Anti-mouse IgD APC-Cy7,BioLegend, Catalog: 405716, validation: https://www.biolegend.com/en-us/products/apc-cyanine7-anti-mouse-igd-6955?GroupID=BLG2055, Statement: FC-Quality Tested
Anti-mouse IgM BV711, BioLegend, Catalog: 406539, validation: https://www.biolegend.com/en-gb/products/brilliant-violet-711-anti-mouse-igm-antibody-18238?GroupID=BLG3548, Statement: FC-Quality Tested
2G12 antibody, NIH AIDS Reagent, Catalog: 1476, validation: This antibody neutralizes a broad variety of SHIV variants and HIV-1 laboratory strains and primary isolates. The epitope is conformational and carbohydrate-dependent. It is directed against N-linked glycans in the C2, C3, V4, and C4 domains of gp120.
3074 antibody, NIH AIDS Reagent, Catalog: 12040, validation: This antibody binds by ELISA to V3 peptides derived from Clade A, B and C viruses and neutralizes viruses from various clades.
PGT145 antibody, NIH AIDS Reagent, Catalog: 12703, validation: https://www.hivreagentprogram.org/Catalog/HRPMonoclonalAntibodies/ARP-12703.aspx
PGT128 antibody, NIH AIDS Reagent, Catalog: ARP-13352, validation: https://www.hivreagentprogram.org/Catalog/HRPMonoclonalAntibodies/ARP-13352.aspx
F425 antibody, NIH AIDS Reagent, Catalog: ARP-7626, validation: ARP-7626 reacts with the base of the V3 loop of gp120 and neutralizes primary isolates.
17b antibody, NIH AIDS Reagent, Catalog: ARP-4091, validation: This antibody binds to a CD4-induced (CD4i) discontinuous epitope on gp120 by ELISA.
10-1074 antibody, NIH AIDS Reagent, Catalog: ARP-12477, validation: https://www.hivreagentprogram.org/Catalog/HRPMonoclonalAntibodies/ARP-12477.aspx
VRC34.01 antibody, CreativeBioLabs, Catalog: N123-VRC34.01, validation: https://www.creativebiolabs.net/anti-hiv-1-gp41-recombinant-antibody-clone-vrc34-01-n123-vrc34-01-82836.htm

Eukaryotic cell lines

Policy information about cell lines

Cell line source(s)	293T (ATCC® CRL-3216™), Expi293F (ThermoFisher), TZM-BL cells (NIH AIDS Reagent, ARP-8129)
Authentication	Cell lines used in this study were not authenticated.
Mycoplasma contamination	The cell lines were tested for mycoplasma contamination by American Type Cell Culture but once received in-house they were not tested.
Commonly misidentified lines (See ICLAC register)	No commonly misidentified cell lines were used in this study.

Animals and other organisms

Policy information about studies involving animals; ARRIVE guidelines recommended for reporting animal research

Laboratory animals	8 week old female BALB/c mice were purchased from Jackson or Charles River Laboratories and housed in the Wistar animal facility. Mice were housed at an ambient temperature of 20-23°C and 45-65% relative humidity on a 12hr/12hr light/dark cycle with 15 minutes dusk and dawn transition periods under Biosafety Level (BSL) I condition in individually ventilated cages and had access to nesting material as well as enrichment (spinning wheel).
Wild animals	The study did not involve wild animals.
Field-collected samples	The study did not involve field-collected samples.
Ethics oversight	All mice were housed in compliance with the NIH and Wistar's Institutional Animal Care and Use Committee guidelines under IACUC protocol 201214.

Note that full information on the approval of the study protocol must also be provided in the manuscript.

Flow Cytometry

Plots

Confirm that:

☒ The axis labels state the marker and fluorochrome used (e.g. CD4-FITC).

☒ The axis scales are clearly visible. Include numbers along axes only for bottom left plot of group (a 'group' is an analysis of identical markers).

☒ All plots are contour plots with outliers or pseudocolor plots.

☒ A numerical value for number of cells or percentage (with statistics) is provided.

Methodology

Sample preparation	Mouse splenocytes were isolated and stimulated with peptides as described in the methodology.
Instrument	BD™ LSR II
Software	FlowJo version 9
Cell population abundance	Cells collected after sorting accounted for about 1/1000 of the total number of cells sorted. Purity of the cells was otherwise not further determined prior to single cell RNA seq analysis (for VH,VL sequencing).
Gating strategy	For our sorting experiment (Figure 5D), stained mouse lymphocytes or splenocytes were first gated on FSC-H vs FSC-A, singlet population was then analyzed with FSC-A and SSC-A, and lymphocyte population (Figure 5D) was selected. Lymphocytes were further gated on cells with no live-dead uptake (Pac Blue negative cells), and CD19-PE-Cy7 positive cells were selected. IgD (APC-Cy7) and IgM (BV711) negative cells were then selected, and MD39 PE and MD39 FITC double positive populations were subsequently sorted for downstream analysis.<br>For intracellular cytokine staining (Supplemental Figure S2), stained mouse l splenocytes were first gated on FSC-H vs FSC-A, singlet population was then analyzed with FSC-A and SSC-A, and lymphocyte population was selected. Lymphocytes were further gated on cells with no live-dead uptake (Pac Blue negative cells) and are CD3 PE-Cy5 positive. These cells were then gated on CD4 (BV510) and CD8 (APC-Cy7). CD3+CD4+ or CD3+CD8+ populations were then analyzed with IFNg-APC uptake to determine IFN-g expression in respective population.

☒ Tick this box to confirm that a figure exemplifying the gating strategy is provided in the Supplementary Information.
